# Telomere length as a marker of changes in body composition and fractures-an analysis of data from the NHANES 2001-2002

**DOI:** 10.3389/fimmu.2023.1181544

**Published:** 2023-09-08

**Authors:** Youfeng Guo, Haihong Zhao, Feng Wang, Haowei Xu, Xiaowei Liu, Tao Hu, Desheng Wu

**Affiliations:** Department of Spine Surgery, Shanghai East Hospital, School of Medicine, Tongji University, Shanghai, China

**Keywords:** body composition, telomere length, fracture, NHANES database, bone mineral density, bone mineral content

## Abstract

**Purpose:**

There has been an association between changes in body composition, fracture incidence, and age in previous studies. Telomere length (TL) has been proposed as a biomarker of aging. However, the relationship between body composition, fractures, and TL has rarely been studied. Therefore, this study aimed to investigate the correlation between TL and body composition and fractures.

Patients and methods: 20950 participants from the 2001-2002 National Health and Nutrition Examination Survey (NHANES) were included in the final analysis. In NHANES, body compositions were measured with DXA, and TL was determined with quantitative PCR. Correlation analysis of TL and body composition was conducted using multivariate weighted linear regression and logistic regression models.

**Results:**

The results showed that TL positively correlated with bone mineral density (BMD) and bone mineral content (BMC) in most body parts. However, BMD and BMC were negatively connected with TL in the upper limbs and skull. Fat content was negatively associated with TL, while muscle content was positively linked to TL. In addition, TL’s trend analysis results were consistent with the regression model when transformed from a continuous to a classified variable. An increase in TL was associated with a higher incidence of wrist fractures, while a decrease in spine fractures. The above correlation also has a certain degree of sex specificity.

**Conclusion:**

Our study indicate that TL is associated with body composition as well as fractures, but further research is needed to confirm these contrasting associations in the skull, upper limbs, and wrists.

## 1 Introduction

Globally, osteoporosis has become more prevalent and a major public health issue with aging populations. About 100,000 Americans over the age of 50 have osteoporosis, and another 340,000 are at risk of developing the disease (1). In 2017-2018, the age-adjusted prevalence of osteoporosis in adults aged 50 years or older was 12.6% (2). It is estimated that there are approximately 20,000 osteoporotic fractures in the United States each year, resulting in up to $100 million in direct health costs (3). It is estimated that 10.3% of non-hospitalized adults aged 50 or above in the United States suffered from osteoporosis in 2010 (4), and the prevalence of osteoporosis among middle-aged and elderly people is still increasing year by year (5, 6). In addition, osteoporosis is a systemic bone disease characterized by bone mass loss, deterioration of bone tissue microstructure, increased bone fragility, and increased fracture risk (7). Osteoporotic fractures not only reduce the quality of life of individuals, but also place a social and economic burden on health care systems all over the world (8). Obviously, the clinical consequences and economic burden of osteoporosis require measures to assess high-risk groups so that prevention and early intervention can be carried out when appropriate. It is reported that many risk factors are related to osteoporotic fractures, including low peak bone mass, hormone factors, the use of certain drugs (such as glucocorticoids), smoking, low physical activity, low calcium and vitamin D intake, race, and personal or family fracture history (9). It is helpful to determine which patients will benefit from an intervention based on clinical assessment of osteoporosis risk factors and objective measurement of bone mineral density (BMD), thereby reducing the mortality rate and incidence rates associated with osteoporotic fractures. Thus, more and more attention is being paid to find novel risk factors and biomarkers to assess osteoporosis risk, which should lead to the development of novel prevention techniques.

More and more studies believe that aging is the biggest risk factor for age-related chronic diseases such as atherosclerosis, tumor, neurodegenerative disease, DM (diabetes mellitus), and osteoporosis (10). Cell senescence is an irreversible growth stagnation phenomenon that occurs in all organisms, and it is the basis for organism senescence. Aging will lead to bone loss (11), which is exacerbated by the reduction of estrogen in postmenopausal women. The lack of estrogen and androgen will weaken the antioxidant stress and autophagy ability of osteoblasts and osteocytes, and make them more vulnerable to oxidative stress (12). At the same time, the number of bone cells in the aging body will also decrease and apoptosis will increase, leading to bone resorption greater than bone formation and bone mass reduction (13). With increasing age, body composition may also change, including a decrease in fat-free tissue mass and an increase in fat mass, leading to an increase in body fat percentage. As an example, body fat increases steadily for most people from the age of 20 to 25 until about 65 (14). More importantly, fat redistributes to the abdomen and internal organs, as well as penetrates into muscles and bones. In contrast, muscle and bone tissue decrease with age.

Telomere, as an aging marker, plays an instrumental role in regulating cell senescence and has received considerable attention in recent years (15). A telomere is a 6-base pair repeat DNA sequence at the end of each chromosome and represents an evolutionarily conservative sequence of DNA (16). It is a special dynamic nucleoprotein structure and is very helpful to stabilize the structural integrity of chromosomes. With each cell division, telomeres will gradually shorten as 50-200 base pairs are lost during DNA replication, resulting in a progressive shortening of telomere length (TL) (17). In cases where telomeres have been shortened beyond a critical length, cells enter proliferation arrest and then stop dividing into cell senescence or apoptosis, such as programmed cell death. In fact, TL serves as a biological clock that records the life span of cells and organisms. When the telomeres are shorter, the life expectancy of the elderly is shorter, and chronic diseases are more likely to occur (18, 19). For example, telomere shortening and DNA damage response can cause senescence of lung epithelial stem cells and pulmonary fibrosis (20).

Based on baseline data from the NHANES database, this study investigated the relationship between TL, body composition, and fractures, as well as TL’s potential as a marker of aging by considering known and potential confounders.

## 2 Materials and methods

### 2.1 Study subjects

A cross-sectional study, the National Health and Nutrition Examination Survey (NHANES) (2001-2002), provided all participant data. Participants had complete BMD, bone mineral content (BMC), fat content, muscle content, fat percentage, and TL data. Relevant information about all participants was collected by well-trained examiners from extensive family interviews, including demographic data, education level and personal disease history. Participants without complete clinical test results, laboratory data, and demographic information were excluded from the study. As a result, 20,950 people aged 20-79 were included in the study. All participants were informed of the study’s ethical approval and consent was obtained from the National Health Statistics Center’s Ethics Review Committee.

### 2.2 Data collection

TL measurement follows the method published by Cawthon (21). DNA was extracted from peripheral blood using phenol chloroform and stored at -80°C with a concentration of more than 100 ng/μl. Then, this method uses quantitative real-time polymerase chain reaction (qRT-PCR) technology. The T/S ratio (Ct (telomere assay)/Ct (single copy gene assay)) is used to evaluate the relative length of telomeres, while Ct is the fractional number of cycles that reach the threshold fluorescence level during qRT-PCR. The Centers for Diseases Control conducted a quality control review before linking TL data to NHANES data files. BMD, BMC, fat content, lean content, and fat percentage of skull, limb bones, pelvis, ribs, trunk bones, spine, subtotal body (total excl head), and total body were evaluated by dual energy X-ray absorptiometry (DXA). Additional details of the BMD assessment can be found on the NHANES website. This study included covariates into its analysis due to the potential impact of them on bone metabolism. A number of covariates were incorporated into this study based on previous studies, including age, gender, race, education level, body mass index (BMI), smoking status, DM, osteoporosis history, past fracture history, physical activity level, and history of pain drugs use (**Supplementary Table S1** for details). Past medical history and lifestyle habits were obtained by questionnaires from the NHANES database.

### 2.3 Statistical analysis

A baseline characteristic is composed of a weighted mean and standard deviation (SD) (continuous variable) and a weighted proportion (categorical variable). The weights used for analysis are selected according to the instructions provided in the NHANES database. In this study, some variables are collected in the mobile examination center (MEC), so we use the exam weight from MEC (WTMEC2YR) for analysis. Second, multivariable weighted linear regression models were employed to evaluate the correlation between TL and body composition. Covariate adjustment is designed for the following four models: Model 1 = unadjusted model; Model 2 = age, gender, race, and BMI; Model 3 = Model 2 + education and smoking; Model 4 = Model 3 + osteoporosis history, DM history, pain drugs intake history, and physical activity level. A β correlation coefficient is calculated between TL and body composition. Third, bivariate multivariable weighted logistic regression was used to evaluate the correlation between TL and fracture events. In addition, the subgroup analysis was also conducted on stratified factors, including age (ten-year age groups constructed based on the participants’ age at survey to enable age-standardized comparisons) and BMI (normal [BMI < 25 kg/m^2^], overweight [25 kg/m^2^ ≤ BMI < 30 kg/m^2^], obesity [BMI ≥ 30 kg/m^2^]). Tests for interaction were performed with likelihood ratio tests. Statistical significance was determined by P values less than 0.05. P-values were corrected for multiple comparisons using Bonferroni’s correction.

## 3 Results

### 3.1 Baseline characteristics

**Figure 1:**
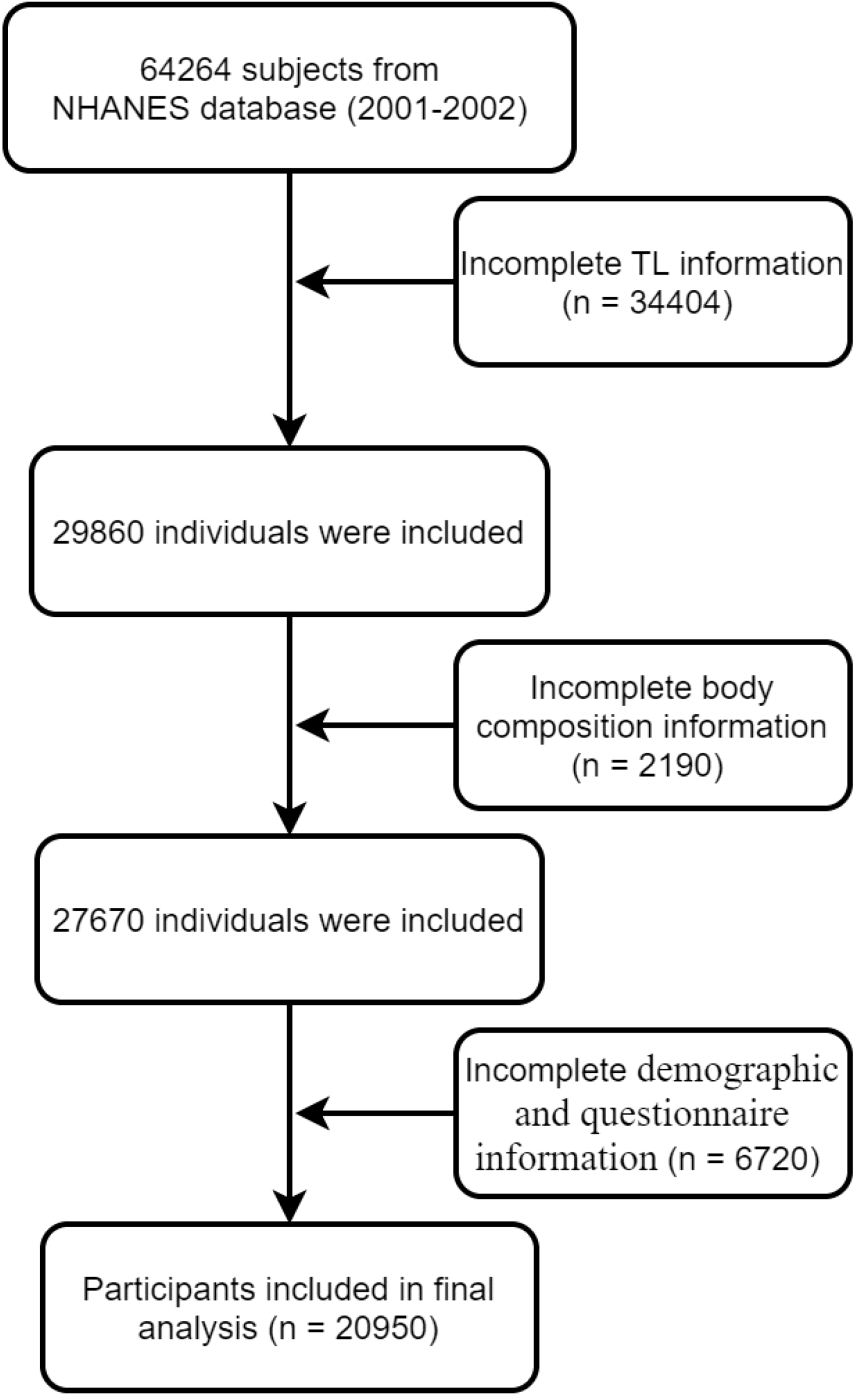
Flowchart of participant selection.

We extracted data from the NHANES (2001-2002) database for 64,264 participants. A flow chart showing how participants are selected is shown in **Figure 1**. To begin with, subjects without TL data (n = 34404) were removed from this study. We also excluded 2190 subjects (n = 2190) with no BMD data and without other body composition information. A third group of subjects (n = 6720) was excluded because they did not have clinical demographics such as their BMI and questionnaire information. A total of 20,950 subjects were analyzed in the final analysis.

The weighted mean age of the population included in the final analysis was 42.04 ± 14.78 years in **Table 1**. There were 11,880 males and 9,070 females, with weighted ratios of 55.3% and 44.7%, respectively. The weighted BMI of the included population was 27.03 ± 5.45 kg/m^2^. About 12.5 percent said they had a broken wrist and 310 had a broken spine. A few comorbidities of the population were recorded through questionnaires, for example, DM and osteoporosis were found in 1190 and 700 people, respectively. The weighted TL for the overall population was 1.12 ± 0.28 bp. It is worth mentioning that correlation analysis showed that TL was significantly associated with age, height, weight, BMI, race, education level, smoking, physical activity level, pain drugs, osteoporosis, and DM, and that sex-based subgroup analysis showed no significant difference (**Supplementary Table S2**). Other baseline characteristics of the sample population, such as race and educational attainment, are shown in **Table 1**. The average BMDs for the head, left arm, left leg, right arm, right leg, left ribs, right ribs, lumbar spine, pelvis, trunk, subtotal body, and total body are 2.23 g/cm^2^, 0.81g/cm^2^, 1.23 g/cm^2^, and 0.83 g/cm^2^, 1.24 g/cm^2^, 0.68 g/cm^2^, 0.67 g/cm^2^, 0.89 g/cm^2^, 1.06 g/cm^2^, 1.31 g/cm^2^, 0.96 g/cm^2^, 1.04 g/cm^2^, 1.16 g/cm^2^, accordingly. For more information regarding the fat and muscle content of different parts of the body, see **Table 2**. In addition, clinical demographic characteristics and body composition were significantly different across TL groups (**Supplementary Tables S3**, **S4**).

**Table 1:** Baseline characteristics of included participants.

Characteristics		Mean or proportion
Age [year], mean (SD)		42.04(14.78)
Gender, n (%)		
	Male	11880(55.3)
	Female	9070(44.7)
Weight [kg], mean (SD)		79.65(18.65)
Height [cm], mean (SD)		171.28(9.84)
BMI [kg/m^2^], mean (SD)		27.03(5.45)
Race, n (%)		
	Mexican American	3100(4.4)
	Other Hispanic	755(4.6)
	Non-Hispanic White	13940(81.8)
	Non-Hispanic Black	2530(5.5)
	Others	625(3.8)
Education, n (%)		
	Under high school	2935(8.7)
	High school or equivalent	4500(22.1)
	Above high school	13515(69.1)
Smoking, n (%)		
	Yes	9420(44.1)
	No	11525(55.9)
Physical activity level, n (%)		
	Moderate	10895(51.4)
	Vigorous	10055(48.6)
Pain drugs, n (%)		
	Yes	5780(27.0)
	No	15170(73.0)
SBP, mean (SD)		119.13(15.81)
DBP, mean (SD)		72.16(11.56)
Osteoporosis, n (%)		
	Yes	700(2.7)
	No	20195(97.1)
DM, n (%)		
	Yes	1190(3.9)
	No	19495(95.3)
Wrist fracture, n (%)		
	Yes	2425(12.5)
	No	18525(87.5)
Spine fracture, n (%)		
	Yes	310(1.6)
	No	20640(98.4)
TL, mean (SD)		1.12(0.28)

BMI, body mass index; DM, diabetes mellitus; DBP, diastolic blood pressure; SBP, systolic blood pressure; TL, telomere length.

**Table 2:** Body composition of included participants.

Index		Mean (SD)
Head		
	BMC (g)	506.99(83.67)
	BMD (g/cm^2)	2.23(0.32)
	Fat (g)	1135.42(164.38)
	Lean (g)	3155.21(437.76)
	Fat percent	23.63(0.58)
Left arm		
	BMC (g)	194.41(50.10)
	BMD (g/cm^2)	0.81(0.10)
	Fat (g)	1507.99(683.96)
	Lean (g)	3077.55(1061.63)
	Fat percent	31.87(11.00)
Left leg		
	BMC (g)	480.26(116.03)
	BMD (g/cm^2)	1.23(0.15)
	Fat (g)	4505.60(1900.21)
	Lean (g)	8330.15(2119.02)
	Fat percent	33.50(9.62)
Right arm		
	BMC (g)	205.17(52.84)
	BMD (g/cm^2)	0.83(0.10)
	Fat (g)	1578.97(698.85)
	Lean (g)	3221.49(1083.99)
	Fat percent	31.81(10.60)
Right leg		
	BMC (g)	481.48(114.01)
	BMD (g/cm^2)	1.24(0.15)
	Fat (g)	4644.80(1953.95)
	Lean (g)	8439.50(2157.38)
	Fat percent	33.90(9.72)
Left rib		
	BMC (g)	81.54(19.28)
	BMD (g/cm^2)	0.68(0.08)
Right rib		
	BMC (g)	85.55(22.87)
	BMD (g/cm^2)	0.67(0.08)
Thoracic spine		
	BMC (g)	128.43(28.11)
	BMD (g/cm^2)	0.89(0.11)
Lumbar spine		
	BMC (g)	61.75(15.16)
	BMD (g/cm^2)	1.06(0.15)
Pelvis		
	BMC (g)	278.59(81.03)
	BMD (g/cm^2)	1.31(0.18)
Trunk		
	BMC (g)	635.86(142.34)
	BMD (g/cm^2)	0.96(0.12)
	Fat (g)	12401.91(5739.73)
	Lean (g)	25863.04(5880.45)
	Fat percent	30.89(8.45)
Subtotal		
	BMC (g)	1997.18(454.90)
	BMD (g/cm^2)	1.04(0.12)
	Fat (g)	24639.27(10292.40)
	Lean (g)	48931.73(11963.83)
	Fat percent	32.14(8.61)
Total		
	BMC (g)	2504.17(488.26)
	BMD (g/cm^2)	1.16(0.11)
	Fat (g)	25774.69(10350.70)
	Lean (g)	52086.94(12337.63)
	Fat percent	31.65(8.15)

BMC, bone mineral content; BMD, bone mineral density.

### 3.2 Association between TL and BMC

**Table 3** shows the correlation between BMC and TL in different parts of the body. The BMC of the lower extremities, left ribs, pelvis, trunk, subtotal body, and total body were associated with increased TL without controlling for covariates (Model 1), while BMC decreases in the left arm, right arm, thoracic spine, and lumbar spine were associated with increased TL. In addition, when adjusted for age, sex, race, and BMI (Model 2), increases in BMC in the lower extremities, left and right ribs, thoracic spine, trunk, subtotal body, and total body were associated with increases in TL, but not in the head and lumbar spine. In addition, when adjusted for education level and smoking (Model 3) or all covariates (Model 4), the results were basically consistent with Model 2. It is worth mentioning that when the left and right ribs and limbs were combined, there was a significant positive correlation between BMC and TL (**Supplementary Table S5**). At the same time, when TL was transformed from a continuous variable to a categorical variable (Q1-Q4), the sensitivity analysis results were consistent with the preliminary analysis results in **Table 4**. Sensitivity analysis indicated that for lower limbs, pelvis, trunk, subtotal body and total body, the higher TL quarter group had higher BMC than the lower TL quarter group. Though the BMC of both upper limbs showed no negative correlation with TL in other models except Model 1 in **Table 3**, sensitivity analyses indicated a downward trend with higher TL. Additional details of the sensitivity analysis are shown in **Table 4**.

**Table 3:** Association between TL and BMC.

Location	Model 1		Model 2		Model 3		Model 4	
	β	95%CI	β	95%CI	β	95%CI	β	95%CI
Head	0.012	-0.552-7.534	-0.016	-8.977–0.367	-0.025	-11.668–3.049	-0.023	-11.153–2.524
Left arm	-0.041	-9.784–4.946	-0.004	-2.247-0.812	-0.003	-2.071-1.001	-0.004	-2.315-0.766
Left leg	0.055	17.182-28.380	0.068	24.060-32.131	0.059	20.479-28.510	0.058	19.980-28.053
Right arm	-0.033	-8.849–3.744	-0.006	-2.721-0.504	-0.006	-2.833-0.405	-0.009	-3.263–0.012
Right leg	0.049	14.359-25.366	0.059	20.031-27.998	0.050	16.389-24.315	0.048	15.474-23.439
Left rib	0.029	1.030-2.893	0.013	0.166-1.634	0.006	-0.353-1.114	0.001	-0.651-0.824
Right rib	-0.008	-1.795-0.415	0.035	2.040-3.726	0.033	1.812-3.505	0.028	1.474-3.174
Thoracic spine	-0.048	-6.123–3.409	0.026	1.445-3.856	0.019	0.702-3.111	0.023	1.061-3.478
Lumbar spine	-0.024	-2.053–0.588	-0.035	-2.657–1.161	-0.044	-3.129–1.634	-0.040	-2.938–1.438
Pelvis	0.159	42.018-49.751	0.079	19.467-26.149	0.072	17.408-24.098	0.070	16.923-23.648
Trunk	0.081	34.214-47.926	0.054	21.675-32.990	0.046	17.662-28.972	0.045	17.093-28.462
Subtotal	0.043	48.089-92.016	0.048	61.979-93.254	0.041	50.798-82.030	0.039	48.148-79.531
Total	0.042	49.968-97.119	0.042	54.966-90.924	0.034	41.114-76.998	0.033	38.987-75.015

BMC, bone mineral content; TL, telomere length.

Model 1: Unadjusted model.

Model 2: age, gender, race, and BMI were adjusted.

Model 3: age, gender, race, BMI, education and smoking were adjusted.

Model 4: age, gender, race, BMI, education, smoking, osteoporosis, DM history, pain drugs intake history, and physical activity level were adjusted.

**Table 4:** Linear regressions for association between body composition and quartile TL.

Location	Model 1	Model 2	Model 3	Model 4
	β[95%CI]	β[95%CI]	β[95%CI]	β[95%CI]
Head				
BMC	0.018[0.366-2.396]	-0.014[-2.128-0.075]	-0.022[-2.749–0.544]	-0.018[-2.420–0.217]
BMD	0.015[0.0004-0.008]	-0.012[-0.007-0.001]	-0.016[-0.009–0.001]	-0.010[-0.007-0.001]
Fat	-0.121[-19.731–15.771]	-0.008[-2.453-0.008]	-0.015[-3.448–0.996]	-0.015[-3.412–0.950]
Lean	-0.105[-46.412–35.847]	-0.002[-4.177-2.400]	-0.009[-6.928–0.379]	-0.010[-7.125–0.553]
Left arm				
BMC	-0.095[-4.866–3.656]	-0.018[-1.190–0.407]	-0.017[-1.137–0.352]	-0.017[-1.152–0.366]
BMD	-0.035[-0.004–0.002]	-0.013[-0.002–0.0003]	-0.010[-0.002–0.00007]	-0.012[-0.002–0.0001]
Fat	-0.122[-83.085–66.612]	-0.013[-11.779–4.495]	-0.014[-12.099–4.785]	-0.011[-10.338–3.023]
Lean	-0.091[-99.232–73.575]	-0.009[-14.727–2.389]	-0.009[-15.173–2.779]	-0.011[-17.134–4.722]
Left leg				
BMC	-0.007[-2.184-0.632]	0.038[2.879-4.950]	0.030[2.063-4.122]	0.029[2.013-4.078]
BMD	0.045[0.004-0.008]	0.042[0.004-0.007]	0.035[0.003-0.006]	0.036[0.003-0.006]
Fat	-0.028[-70.798–24.703]	0.010[3.000-29.374]	0.006[-3.832-22.568]	0.006[-3.261-23.226]
Lean	-0.023[-68.444–17.034]	0.056[92.215-120.033]	0.048[76.564-104.063]	0.046[72.758-100.292]
Right arm				
BMC	-0.083[-4.569–3.291]	-0.015[-1.133–0.308]	-0.015[-1.144–0.316]	-0.016[-1.190–0.361]
BMD	-0.037[-0.005–0.002]	-0.027[-0.003–0.002]	-0.026[-0.003–0.002]	-0.027[-0.003–0.002]
Fat	-0.125[-86.977–70.151]	-0.010[-9.726–2.489]	-0.011[-10.571–3.306]	-0.007[-8.320–1.069]
Lean	-0.079[-90.093–63.871]	0.001[-4.782-7.518]	-0.0003[-6.456-5.888]	-0.002[-8.604-3.765]
Right leg				
BMC	-0.006[-1.976-0.791]	0.037[2.758-4.801]	0.029[1.932-3.963]	0.028[1.795-3.831]
BMD	0.036[0.003-0.007]	0.027[0.002-0.005]	0.019[0.001-0.004]	0.019[0.001-0.004]
Fat	-0.027[-70.260–22.859]	0.012[6.969-33.975]	0.008[0.043-27.087]	0.008[0.996-28.108]
Lean	-0.016[-57.390–5.043]	0.064[110.230-138.369]	0.056[95.289-123.111]	0.055[91.460-119.303]
Left rib				
BMC	0.014[0.007-0.475]	0.019[0.135-0.510]	0.012[0.015-0.390]	0.008[-0.044-0.333]
BMD	0.039[0.002-0.004]	0.005[-0.001-0.001]	0.002[-0.001-0.001]	-0.001[-0.001-0.001]
Right rib				
BMC	-0.047[-1.233–0.679]	0.013[0.052-0.484]	0.010[-0.004-0.429]	0.007[-0.081-0.353]
BMD	0.039[0.002-0.004]	0.024[0.001-0.003]	0.024[0.001-0.003]	0.024[0.001-0.003]
Thoracic spine				
BMC	-0.082[-2.414–1.734]	0.008[-0.095-0.522]	0.002[-0.258-0.359]	0.006[-0.154-0.464]
BMD	-0.074[-0.009–0.006]	-0.027[-0.004–0.001]	-0.028[-0.004–0.001]	-0.022[-0.004–0.001]
Lumbar spine				
BMC	-0.034[-0.651–0.283]	-0.030[-0.599–0.216]	-0.037[-0.698–0.315]	-0.032[-0.633–0.250]
BMD	0.056[0.006-0.010]	0.001[-0.002-0.002]	-0.004[-0.003-0.001]	0.001[-0.002-0.002]
Pelvis				
BMC	0.113[7.242-9.196]	0.046[2.485-4.200]	0.039[1.977-3.693]	0.039[1.941-3.662]
BMD	0.061[0.008-0.012]	0.006[-0.001-0.003]	0.002[-0.002-0.002]	0.002[-0.002-0.002]
Trunk				
BMC	0.039[3.237-6.689]	0.029[2.289-5.189]	0.022[1.346-4.242]	0.022[1.343-4.248]
BMD	0.060[0.005-0.008]	0.008[-0.001-0.002]	0.004[0.493–0.001]	0.006[-0.001-0.002]
Fat	-0.187[-1029.556–892.721]	-0.028[-173.537–118.535]	-0.031[-187.965–132.952]	-0.030 [-182.884–127.794]
Lean	-0.111[-657.381–515.564]	0.019[62.630-132.357]	0.017[54.115-123.591]	0.015[45.919-115.607]
Subtotal				
BMC	-0.011[-10.116-0.923]	0.024[5.906-13.921]	0.018[3.360-11.359]	0.017[3.108-11.128]
BMD	0.038[0.003-0.005]	0.019[0.001-0.003]	0.014[0.0003-0.003]	0.015[0.0003-0.003]
Fat	-0.131[-1332.669–1085.054]	-0.013[-170.456-76.787]	-0.017[-199.689–106.122]	-0.015 [-189.087–95.272]
Lean	-0.077[-968.550–679.077]	0.030[253.603-387.851]	0.026[212.290-345.924]	0.024 [192.337-326.305]
Total				
BMC	-0.007[-9.140-2.709]	0.020[4.282-13.493]	0.013[1.121-10.307]	0.013[1.198-10.401]
BMD	0.047[0.003-0.006]	0.009[-0.0003-0.002]	0.003[-0.001-0.002]	0.005[-0.001-0.002]
Fat	-0.132[-1351.101–1102.123]	-0.013[-171.828–77.861]	-0.017[-202.045–108.210]	-0.016 [-191.403-97.317]
Lean	-0.078[-1014.186-715.701]	0.029[251.241-388.435]	0.025[207.198.343.709]	0.023 [187.056-323.909]

BMC, bone mineral content; BMD, bone mineral density.

Model 1: Unadjusted model.

Model 2: age, gender, race, and BMI were adjusted.

Model 3: age, gender, race, BMI, education and smoking were adjusted.

Model 4: age, gender, race, BMI, education, smoking, osteoporosis, DM history, pain drugs intake history, and physical activity level were adjusted.

Subgroup analyses of the relationship between BMC and TL stratified by sex, age, BMI, and race are available in **Supplementary Tables S6**, **S7**, **S8**, and **S9**. Results of multiple linear regression model 4 showed an opposite trend in the correlation between skull and lumbar BMC and TL in males and females. In addition, the results also showed that the negative correlation between BMC and TL at lumbar spine mainly existed in women, while that between cranial BMC and TL mainly occurred in men. Further, the BMCs of right ribs were also positively correlated with TL mainly in women. The correlation between TL and BMC at other sites was not significantly different between males and females and was consistent with the preliminary analysis. It was found that BMC in both upper limbs, pelvis, thoracic vertebras, and lumbar vertebras, as well as in ribs and skull were significantly affected by sex and TL interaction. Age-based subgroup analyses showed that TL and BMC were more likely to correlate negatively in people aged 30-40 and 60-70, whereas positive correlations were more common in other age groups (**Supplementary Table S7**). A subgroup analysis based on the BMI of people found that there was a positive correlation between TL and BMC in those with a BMI less than 30kg/m2, whereas negative associations were present in those with a BMI more than 30kg/m2 (**Supplementary Table S8**). At the same time, the interaction between TL and BMI was more significant in the skull, right limbs, thoracolumbar, pelvis, and trunk. There is also a significant difference in the correlation between TL and BMC between different races, i.e., the negative correlation is more prominent among non-Hispanic blacks (**Supplementary Table S9**). In addition, the interaction between TL and race had significant effects on BMC in all areas except lower limbs and right rib. Additional detailed subgroup analysis results are presented in **Supplementary Tables S6**–**S9**.

### 3.3 Association between TL and BMD

The correlation between BMD and TL in different parts of the body is listed in **Table 5**. With no adjustment for covariates (Model 1), increased BMD in the left leg, right leg, left ribs, right ribs, lumbar spine, pelvis, trunk, subtotal body, and total body were associated with increased TL, while decreased BMD in the thoracic spine was associated with increased TL. Additionally, when age, gender, race, and BMI were adjusted for (Model 2), BMD increases were associated with TL increases in the left leg, right leg, right ribs, subtotal body, trunk, and total body, but not with TL increases in the right arm, left arm, and thoracic vertebrae. Subsequently, after adjustment for education level and smoking, BMD in the left leg, right leg, right ribs and subtotal body was correlated with an increase in TL. In contrast, the left arm, right arm and thoracic vertebrae showed an opposite trend. In addition, after adjusting all covariates (model 4), it is consistent with model 3. There is also a significant positive correlation between BMC and TL when left and right ribs and limbs are combined (**Supplementary Table S5**). At the same time, when TL was transformed from a continuous variable to a categorical variable (Q1-Q4), the sensitivity analysis results were consistent with the preliminary analysis results. Although Model 1 failed to show a correlation between BMD and TL in both upper limbs, trend analysis revealed a negative correlation in Model 3, which also occurred in cranial BMD in Model 3. Additional details of the sensitivity analysis are shown in **Table 4**.

**Table 5:** Association between TL and BMD.

Location	Model 1		Model 2		Model 3		Model 4	
	β	95%CI	β	95%CI	β	95%CI	β	95%CI
Head	0.000	-0.015-0.016	-0.006	-0.023-0.008	-0.012	-0.029-0.003	-0.007	-0.024-0.008
Left arm	-0.001	-0.005-0.004	-0.017	-0.009–0.003	-0.014	-0.008–0.002	-0.017	-0.009–0.003
Left leg	0.088	0.040-0.055	0.059	0.026-0.037	0.051	0.022-0.033	0.051	0.022-0.033
Right arm	-0.010	-0.009-0.001	-0.038	-0.017–0.010	-0.037	-0.017–0.010	-0.040	-0.018–0.011
Right leg	0.067	0.028-0.043	0.030	0.010-0.022	0.020	0.005-0.016	0.019	0.004-0.016
Left rib	0.058	0.014-0.022	0.003	-0.003-0.005	0.000	-0.003-0.004	-0.004	-0.005-0.002
Right rib	0.062	0.014-0.022	0.027	0.004-0.012	0.027	0.004-0.011	0.025	0.004-0.011
Thoracic spine	-0.069	-0.034–0.023	-0.030	-0.017–0.007	-0.032	-0.019–0.008	-0.028	-0.017–0.006
Lumbar spine	0.050	0.020-0.035	0.002	-0.006-0.009	-0.003	-0.010-0.006	0.000	-0.008-0.008
Pelvis	0.070	0.037-0.055	0.011	-0.001-0.015	0.007	-0.003-0.013	0.005	-0.005-0.012
Trunk	0.077	0.027-0.038	0.015	0.001-0.012	0.011	-0.001-0.010	0.011	-0.001-0.010
Subtotal	0.070	0.024-0.035	0.025	0.006-0.015	0.020	0.004-0.013	0.018	0.003-0.012
Total	0.067	0.021-0.032	0.014	0.001-0.010	0.007	-0.002-0.004	0.007	-0.002-0.008

TL, telomere length; BMD, bone mineral density.

Model 1: Unadjusted model.

Model 2: age, gender, race, and BMI were adjusted.

Model 3: age, gender, race, BMI, education and smoking were adjusted.

Model 4: age, gender, race, BMI, education, smoking, osteoporosis, DM history, pain drugs intake history, and physical activity level were adjusted.

Subgroup analyses stratified by sex, age, BMI, and race are presented in **Supplementary Tables S6**–**S9**. Results of multiple linear regression models showed an opposite trend in the correlation between head, left ribs, right ribs, and lumbar spine BMD and TL in males and females. Additionally, it was found that the positive correlation between TL and BMD of the right leg, right rib, trunk, subtotal body, and total body mainly exists in males. The correlation between TL and BMD at other sites was not significantly different between males and females. Age-based subgroup analysis showed that positive and negative associations between TL and BMD were consistent with BMC in the predisposition population (**Supplemental Table S7**). Not only that, as with BMC, the negative association between TL and BMD was also found primarily in people with BMIs greater than 30kg/m2 and in non-Hispanic blacks (**Supplemental Tables S8**, **S9**). At the same time, the interaction between TL and BMI, sex, or race was significant for most skeletal BMD. Additional detailed subgroup analysis results are presented in **Supplementary Tables S6**–**S9**.

### 3.4 Association between TL and fat

**Table 6** shows the correlation between body fat content and TL. Without adjustment for covariates (Model 1), increases in the head, upper limbs, lower limbs, trunk, subtotal body, and total body fat content were associated with decreased TL. However, in models 2, 3, and 4, the correlation between lower limb fat content and TL was significantly lost, while the other sites remained unchanged. It is worth mentioning that the percentage of fat in all sites increased with the decrease in TL (*p* < 0.05). In line with this, the percentage of fat in the body increases with age as well. The correlation between fat and TL is not significant when the left and right limbs are combined (**Supplementary Table S5**). Meanwhile, sensitivity analysis showed that the quarter with a higher TL had less fat content than the quarter with a smaller TL for the rest of the body except the lower extremities in **Table 4**. It is significant to mention that according to trend analysis in other models except Model 1, the higher the TL was, the higher the fat content in the lower limbs, which follows the opposite trend from other body parts. Additional details of the sensitivity analysis are shown in **Table 4**.

**Table 6:** Association between telomere length and fat.

Location	Model 1		Model 2		Model 3		Model 4	
	β	95%CI	β	95%CI	β	95%CI	β	95%CI
Head	-0.092	-61.746–45.926	-0.017	-14.779–5.166	-0.024	-19.095–9.511	-0.025	-19.347–9.708
Left arm	-0.133	-357.160–291.638	-0.010	-38.062–9.590	-0.010	-39.726–11.123	-0.007	-31.221–2.553
Left leg	-0.059	-493.616–310.274	0.004	-25.164-77.932	-0.001	-55.694-47.526	0.000	-54.858-48.922
Right arm	-0.136	-372.986–306.068	-0.009	-36.067–7.781	-0.010	-39.815–11.410	-0.006	-29.813–1.400
Right leg	-0.054	-467.476–278.886	0.011	22.677-128.228	0.007	-7.259-98.479	0.007	-7.495-98.736
Trunk	-0.186	-4077.427–3532.308	-0.027	-659.089–444.081	-0.030	-724.563–509.453	-0.028	-683.378–467.466
Subtotal	-0.143	-5736.232–4751.608	-0.013	-678.541-312.454	-0.017	-809.410–443.625	-0.015	-749.044–381.482
Total	-0.143	-5792.809–4802.702	-0.014	-689.094–321.847	-0.017	-824.235–457.407	-0.016	-764.099–395.483

TL, telomere length.

Model 1: Unadjusted model.

Model 2: age, gender, race, and BMI were adjusted.

Model 3: age, gender, race, BMI, education and smoking were adjusted.

Model 4: age, gender, race, BMI, education, smoking, osteoporosis, DM history, pain drugs intake history, and physical activity level were adjusted.

The results of the subgroup analysis of the relationship between fat content and TL stratified by sex, age, BMI, and race are shown in **Supplementary Tables S6**–**S9**. Based on the results of multiple linear regression models, a negative correlation between TL and head, left arm, trunk, subtotal body and total body fat is primarily found in men. The correlation between TL and fat content in the left leg, right hand, and right leg was not significantly different between males and females. TL was positively associated with fat in people over 70 years of age, while negative associations were more common in people of other ages (**Supplemental Table S7**). It was found that people with a BMI of 25 to 30 kg/m2 had a negative association between TL and fat (**Supplement Table S8**). A significant difference was also found in the correlation between TL and fat among different races, with a positive correlation more prominent among non-Hispanic blacks (**Supplemental Table S9**). In addition, the interaction between TL and age, sex, or race has a significant impact on fat. Additional detailed subgroup analysis results are presented in **Supplementary Tables S6**–**S9**.

### 3.5 Association between TL and lean

In **Table 7**, the correlation between muscle content in different body regions and TL is presented. When no covariates were adjusted (Model 1), muscle content of the head, left arm, trunk, subtotal body and total body was negatively correlated with TL, while it was positively correlated with TL for lower limbs. The correlation between double upper limbs muscle content and TL, however, lost significance in models 2, 3, and 4. There was no change in the correlation between muscle content in the skull and TL, while there was a shift to a positive correlation in other parts. It’s important to highlight that upon consolidating the left and right limbs, a notable and statistically significant positive correlation emerged between TL and lean mass, as detailed in **Supplementary Table S5**. At the same time, sensitivity analysis (**Table 4**) results showed that the muscle content of the quarter group with higher TL was higher than that of the quarter group with lower TL in both lower limbs, trunk, subtotal body and total body in other models except Model 1. In contrast, the correlation between TL and muscle content in the left hand and head was opposite. Although left-hand muscle content and TL were not correlated in models 2, 3, or 4, trend analysis demonstrated a negative correlation for these models.

**Table 7:** Association between TL and lean.

Location	Model 1		Model 2		Model 3		Model 4	
	β	95%CI	β	95%CI	β	95%CI	β	95%CI
Head	-0.075	-137.599–95.405	-0.012	-31.448–5.746	-0.020	-43.516–17.918	-0.021	-45.474–19.736
Left arm	-0.038	-194.643–92.104	0.001	-19.498-28.735	0.001	-21.353-27.114	-0.002	-30.392-18.249
Left leg	0.031	131.027-335.744	0.076	519.757-627.950	0.067	453.668-560.729	0.065	434.385-541.844
Right arm	-0.031	-173.599-68.877	0.005	-4.711-43.357	0.003	-11.983-36.278	0.000	-22.314-26.149
Right leg	0.036	175.003-383.388	0.082	577.194-686.628	0.074	512.806-621.111	0.071	491.612-600.274
Trunk	-0.057	-1471.547–904.082	0.033	558.695-830.766	0.031	506.821-778.056	0.029	476.804-749.474
Subtotal	-0.022	-1517.890–361.803	0.045	1662.829-2186.039	0.041	1471.020-1992.222	0.038	1381.178-1904.903
Total	-0.024	-1652.427–460.273	0.043	1638.436-2173.235	0.039	1434.639-1967.166	0.037	1342.883-1877.986

TL, telomere length.

Model 1: Unadjusted model.

Model 2: age, gender, race, and BMI were adjusted.

Model 3: age, gender, race, BMI, education and smoking were adjusted.

Model 4: age, gender, race, BMI, education, smoking, osteoporosis, DM history, pain drugs intake history, and physical activity level were adjusted.

The results of subgroup analysis of the relationship between muscle content and TL stratified by sex, age, BMI, and race are shown in **Supplementary Tables S6**–**S9**. The results of multiple linear regression model 4 showed a negative correlation between head muscles and TL primarily among males, and left upper limb muscles and TL primarily among females. In addition, the muscle content of lower limbs, trunk, subtotal body and total body was positively correlated with TL and there was no significant difference between male and female. A subgroup analysis based on age showed positive and negative correlations between TL and lean in each age group (**Supplementary Table S7**). Subgroup analysis based on human BMI found that TL was positively correlated with lean in most bone sites and appeared in different BMI groups, and only negatively correlated with the skull of people with BMI<25kg/m2 and the double upper limbs of people with BMI ≥ 30kg/m2 (**Supplement Table S8**). In addition, the negative correlation between TL and lean may appear in all races except Mexican-American and is not special (**Supplementary Table S9**).

### 3.6 Association between TL and fracture

A multivariate binary logistic regression model (**Table 8**) based on clinical parameters revealed that each unit increase in proven age (OR: 1.023; 95%CI: 1.020-1.026), an education level (OR: 0.793; 95%CI: 0.743-0.847), smoking history (OR: 1.298; 95%CI: 1.188-1.419), use of pain drugs (OR: 2.675; 95%CI: 2.432-2.942), history of DM (OR: 0.809; 95%CI: 0.712-0.920), BMD (OR: 16.436; 95%CI: 10.363-26.067) was significantly associated with wrist fractures. However, TL (OR: 1.017; 95%CI: 0.853-1.213) did not significantly affect the incidence of wrist fractures. Interestingly, trend analysis results showed that the higher TL quartile had a higher incidence of wrist fracture than the lower TL quartile (*p* < 0.001) in **Table 9**. On the other hand, the multivariate binary logistic regression model showed that age increased by one unit (OR: 0.989; 95%CI: 0.981-0.997), BMI (OR: 0.974; 95%CI: 0.953-0.995), race (OR: 1.226; 95%CI: 1.071-1.404), smoking history (OR: 1.506; 95%CI: 1.192-1.902), history of pain drugs use (OR: 1.583; 95%CI: 1.235-2.029), history of DM (OR: 0.760; 95%CI: 0.601-0.961), physical activity level (OR: 2.056; 95%CI: 1.599-2.644), BMD (OR: 21.415; 95%CI: 6.816-67.285), TL (OR: 0.343; 95%CI: 0.221-0.532) was identified as an independent predictor of spine fracture. At the same time, trend analysis (**Table 9**) indicated that the higher TL quartile had a lower incidence of spine fracture than the lower TL quartile (*p* < 0.001). In addition, logistics regression based on gender stratification (**Supplementary Table S10**) suggests that TL is not an independent influencing factor for wrist fracture in male or female indicators. At the same time, it also suggests that TL has a significant effect on the occurrence of spine fracture mainly in the male population. Next, we examined the relationship between TL and fracture by gender stratification (**Supplementary Table S11**). For men, the quartile with the greater TL had a reduced incidence of spinal fractures than the quartile with the inferior TL. Interestingly, however, the higher TL quartile did not simply show a higher incidence of wrist fractures than the lower TL quartile in **Supplementary Table S11**. The group with quartile 1 and quartile 4 of TL had a higher wrist fracture rate than that with quartile 2 and quartile 3. For women, the higher TL quartile had a higher incidence of wrist fractures than the lower TL quartile. Meanwhile, the group in quartile 3 had a higher fracture rate of the spine than the group in quartile 4. Through age-based subgroup analysis (**Supplementary Table S12**), it became evident that concerning spinal fractures, elevated TL correlated with reduced fracture risk among individuals aged 40-50 and 60-80 years; conversely, a contrasting trend emerged within the 30-40 age group. Turning to wrist fractures, a contrasting pattern was observed: heightened TL corresponded to an elevated fracture risk among those aged 20-40 and 70-80 years, whereas individuals within the 50-70 age range exhibited an inverse relationship between TL and fracture risk. Further, BMI-based subgroup analyses revealed that the higher the TL for people with BMI < 30kg/m2, the higher the wrist fracture incidence and the lower the spinal fracture incidence. The opposite was true for people with BMI ≥ 30kg/m2 (**Supplementary Table S12**).

**Table 8:** Association between TL and fracture.

Variable	Wrist fracture		Spine fracture	
	OR	95%CI	OR	95%CI
Age	1.023	1.020-1.026	0.989	0.981-0.997
Gender	1.757	1.591-1.940	1.938	1.485-2.529
BMI	1.003	0.995-1.012	0.974	0.953-0.995
Race	1.011	0.961-1.064	1.226	1.071-1.404
Education	0.793	0.743-0.847	0.912	0.779-1.068
Smoking	1.298	1.188-1.419	1.506	1.192-1.902
Pain drugs	2.675	2.432-2.942	1.583	1.235-2.029
DM	0.809	0.712-0.920	0.760	0.601-0.961
Physical activity	0.979	0.895-1.070	2.056	1.599-2.644
Total BMD	16.436	10.363-26.067	21.415	6.816-67.285
TL	1.017	0.853-1.213	0.343	0.221-0.532

TL, telomere length; BMI, body mass index; DM, diabetes mellitus; BMD, bone mineral density.

**Table 9:** The trend analysis of the association of TL with wrist fracture and spine fracture.

Variable	Case	Wrist fracture		Spine fracture	
		OR [95%CI]	P for trend	OR [95%CI]	P for trend
TL (median[range])					
Q1(0.811[≤ 0.898])	5255	0.954[0.823-1.105]	< 0.001	1.914[1.391-2.635]	< 0.001
Q2(0.976[0.898-1.053])	5225	0.687[0.603-0.783]		1.941[1.423-2.647]	
Q3(1.131[1.053-1.220])	5235	0.523[0.462-0.591]		4.975[3.196-7.745]	
Q4(1.351[> 1.220])	5235	Reference		Reference	

TL, telomere length.

## 4 Discussion

We analyzed data from the National Health and NHANES database on the relationship between TL, BMD, BMC, muscle content, and fat content (2001-2002). To the best of our knowledge, it is the largest investigation to date into the relationship between TL and body composition and fractures. We performed this analysis in a large, population-based study, taking into account a variety of lifestyle/demographic and medical factors known to influence TL. Results showed that TL positively correlated with BMD, BMC, and muscle content in most parts of the body, and negatively correlated with fat content. Notably, we also observed a negative correlation between TL and thoracic BMC in the unadjusted model. However, no correlation was observed between TL and thoracic BMC after adjusting for relevant variables. In stark contrast, TL was negatively associated with thoracic BMD in both adjusted and unadjusted models. In addition, we found a significant or nearly significant negative correlation between BMC, BMD, and TL at three anatomical sites (cranium, and upper limbs). At the same time, the above correlation has a certain degree of sex specificity. We also found a modest association between higher TL and a lower chance of spine fractures and a higher chance of wrist fractures regardless of confounding factors.

As a marker of aging, does TL correlate with bone mass changes? In this study, TL was observed to be correlated with BMD, BMC, fat content, and muscle content. Wang et al. revealed that telomere dysfunction can cause osteoblast differentiation to decrease in mice, resulting in musculoskeletal dysfunction and accelerated aging (22). This evidence suggests that telomere wear may lead to stronger bone resorption than bone formation, leading to osteoporosis in the elderly. The relation between TL and markers of bone turnover is, however, limited in epidemiological studies. As of today, there is also not much evidence to support a causal link between TL and osteoporosis. Previous studies have shown that shorter telomeres are associated with tissue inflammation (23). In fact, higher levels of oxidative stress can accelerate telomere shortening (24). These results seem to be consistent with the hypothesis that inflammation is associated with the pathogenesis of osteoporosis and telomere shortening. It is well known that telomere erosion is enhanced by oxidative stress during each replication cycle (25). Thus, TL may partly record an individual’s lifetime cumulative burden of oxidative stress and inflammation cytokines, both of which are associated with decreased BMD. Bekaert et al. found that shorter TL was associated with forearm bone loss (26). Moreover, a Chinese population-based study detected a correlation between higher TL and higher femoral neck BMD, although this association seemed to diminish with age (27). Conflicting reports have been found regarding the effect of TL on osteoporosis risk. A large study has shown that TL is not associated with hip BMD, osteoporosis, or fracture in the elderly population (28). Furthermore, TL does not appear to be associated with baseline BMD or hip BMD changes in a study from China (27). These findings do not necessarily negate the concept of TL as a biological marker of bone aging, as these studies did not cover a wide age range, and BMD at the hip may be more affected by mechanical stress than at other body sites (29). Moreover, the regulation of TL is complex, involving genetics and lifestyle factors (30), resulting in inconsistent results across these studies because different study designs, TL measurements, and statistical methods were used in the analysis. According to Kveiborg et al., osteoblast telomeres *in vitro* shorten with age, but there is no significant difference in TL between osteoporosis patients and healthy individuals, suggesting that osteoporosis patients may not experience widespread premature cellular aging (31). However, it’s worth considering that although there have been studies demonstrating that TL decreases with age, the magnitude of this effect seems small compared to the differences between individuals. So how important are changes in TL as a key process of aging?

In addition, this study observed differences in the correlation between TL and BMD at different bone sites. For example, TL was negatively correlated with skull BMD but positively correlated with lower limbs BMD. While the exact mechanism is uncertain, there are some possible explanations. First, we speculated that the difference in bone sites might be due to age or BMI. The BMD at different bone sites was compared in groups of varying ages or BMI to accomplish this. According to the results (**Supplemental Table S13**), the BMD and BMC of older adults were significantly lower than those of younger adults (*p* < 0.001). The BMD and BMC at any bone site were considerably higher in people with higher BMI than those with lower BMI (**Supplemental Table S14**, *p* < 0.001). Thirdly, there is a great deal of variation in the bone structure of the different parts of the bone. In addition, there were significant differences between different bone sites at the genetic and cellular levels, suggesting that different bone sites may respond differently to inflammatory stimuli (32). Age-related changes were observed in the microstructures of the femur, tibia, and vertebral bones, but the microstructure of interparietal bones and mandibles, which develop via intramembranous ossification, was less affected by age and gender (33). This may be due to the difference in ossification mechanisms involved in bone development: intramembranous ossification of craniofacial bones *vs*. endochondral ossification of almost all trunk bones. The endomembranous bone is mainly located in the parietal bone, the skull base, the facial bone, the clavicle, etc. Cartilaginous internalized bone can be found in limbs, spines, and pelvises (34). Studies have shown that differences in ossification patterns lead to differences in bone matrix composition, osteogenic differentiation, and cell proliferation (35–37). It was interesting to observe that the long bone showed a larger osteoclast size and a higher rate of bone renewal than the trabecular bone of the skull (32). Therefore, the evidence from previous studies supports our hypothesis to a certain extent, and further research is also needed to answer this question. Our results also show that higher TL is positively associated with wrist fractures and negatively associated with spinal fractures. In addition to the above reasons, we speculate that it may also be related to life habits. As aging progresses, many older people also complain of mobility problems, ranging from complete bed rest to difficulty walking or traveling, with relatively high levels of movement of the hands due to the need to eat. Furthermore, this research revealed a race-specific correlation between TL and BMD, suggesting that the connection between the two factors varies among different ethnic groups. Several factors contribute to this phenomenon: 1. Genetic Factors: Genetic diversity across races influences bone tissue growth and metabolism. Unique genetic variations distributed among populations can impact bone density. For example, Medina et al. discovered a positive association between specific alleles and BMD in populations with sub-Saharan African heritage (38). This study indicated that children of sub-Saharan African descent exhibit elevated BMD, even after accounting for shared lifestyle influences between mothers and children. These variations may result from selective pressures on the African continent. Moreover, our study identified that individuals of Non-Hispanic Black ethnicity, with African ancestry, demonstrate elevated BMD and BMC levels in comparison to other racial groups. Geographical factors, such as sunlight exposure and climate conditions, could further contribute to varying bone density among ethnicities. 2. Dietary Patterns: Ethnic-specific dietary habits influence the intake of essential nutrients, including vitamin D, crucial for bone health. This variance in nutrient consumption subsequently affects bone density. Additionally, lifestyle behaviors, such as physical activity and outdoor engagement, diverge across ethnic groups and contribute to disparities in BMD levels (39).

Our study showed an opposite trend in the correlation between head, left rib, and lumbar BMD and TL in males and females. In addition, the TL and BMD of the right leg, right rib, trunk, subtotal body, and total body tend to be positively correlated mainly in males. A negative correlation was also observed between thoracic BMD and TL predominantly in men. There was no significant difference between males and females in the correlation between TL and BMD at other sites. Thus, the association between TL and body composition is sex-specific, and this may be caused by hormone levels, especially sex hormones. The drop in endogenous estrogen production after menopause may lead to decreased bone mass, altered inflammatory status, and immune response (40). Estrogen has been shown to induce telomerase (41), while androgens directly or indirectly cause the opposite effect (42). Therefore, estrogen may be associated with sex differences in osteoporosis. Estrogen could increase trabecular and cortical bone (43). It is worth noting that trabecular bone and cortical bone are influenced differently by estrogen therapy over a long period of time, with different proportions of trabecular bone being affected (44). However, testosterone increases the structural and mechanical properties of trabecular bone, but decreases most of these properties of cortical bone (45). In addition, studies have shown that estrogen decreases and androgens increase cortical bone size, leading to well-known sexual dimorphism in cortical bone geometry (46). Nevertheless, sex steroids may not always be harmful to the telomeres, as some studies have not found a statistically significant link between them and TL in older men. Nielsen et al. found no significant association between TL and BMD despite considering the interactions between TL and age and menopausal status (47). The prevalence of osteoporosis and fractures in women is higher than in men, according to previous studies (48, 49), and that men have shorter telomeres than women, resulting in shorter telomeres than women (50). However, there was actually no sex difference in TL at birth (51).

Adipose tissue is thought to be an efficient system for storing energy. However, there is substantial evidence that adipose tissue does not remain constant throughout a mammal’s life (52). From an evolutionary perspective, the increase in fat content early in life during pregnancy and breastfeeding is a beneficial strategy to prevent energy shortages (53). There is no consensus on the evolutionary significance of fatty tissue deposition during aging, the third peak of fatty tissue deposition. A number of studies have demonstrated an increase in fat mass and a decrease in fat-free mass as we age (54, 55). In the study, we found a negative correlation (*p* < 0.05) between TL and fat percentages across all sites, while a positive correlation between age and fat percentages (*p* < 0.05). In addition, a gender difference was observed, with significantly higher fat gain in women. According to multiple linear regression models, our study found a negative correlation between TL and head, trunk, subtotal body, and total body fat primarily among men. Between males and females, there was no statistically significant difference in TL correlation with fat content in the left leg, right hand, and right leg. It has been suggested that girls have a slight increase in absolute fat mass during puberty, while boys do not, which could indicate an adaptation related to reproduction. Abdominal obesity continued to increase with age in both men and women, but the rate of weight and fat gain decreased over time, especially among men (56). Our results showed that older people had a relatively higher fat content than younger people (*p* < 0.05; **Supplementary Table S13**). On the other hand, the amount of fat tissue varies greatly between different parts of the body, with a proportionately larger increase in the extremities compared to the trunk (55). Kuhlow et al. found that telomerase-deficient young adult mice exhibited impaired glucose tolerance without changes in body fat content, energy expenditure, and insulin sensitivity (57). This latter observation conflicts with previous reports that telomere shortening in humans is associated with insulin resistance (58). Interestingly, insulin is a key regulator of energy metabolism, including fat storage. In addition, an imbalance between the release and oxidation of free fatty acids (FFA) has been hypothesized, with a decrease in fat oxidation possibly due to a decrease in oxidized tissue mass (58, 59). Furthermore, other studies have found that older individuals have lower levels of fat oxidation (60). Apart from the above, one of the factors contributing to body fat may be the decline in hormones associated with aging, such as testosterone, estrogen, and growth hormone (61). The body composition of older men is influenced by a relative decrease in serum testosterone and postmenopausal fat deposits are higher than those of premenopausal women (62).

With the occurrence of aging, inflammation and oxidative stress are exacerbated, mitochondrial dysfunction increases and muscle cell regeneration is inhibited (63, 64). A longitudinal study revealed significant reductions in muscle strength and total muscle area with aging in the enrolled population (65). Similarly, our study also demonstrated that the muscle content of elderly people was significantly lower than that of young people (**Supplementary Table S13**, *p* < 0.05). However, previous studies have shown inconsistent results between TL and sarcopenia. A mendelian randomization study including 261,000 older individuals failed to show an association between TL and muscle content (66). Also, Woo et al. failed to find significant differences between sarcopenia and non-sarcopenic patients in TL (67). In contrast, Marzetti et al. found a significant association between TL and muscle content (68). Studies have also shown that women lose muscle more slowly than men due to their higher rate of muscle protein synthesis and translation (69, 70), which may explain why women lose muscle more slowly as they age. According to our study, head muscles are inversely related to TL primarily among males, while left upper limb muscles are inversely related to TL primarily among females. It is mainly the male population who showed a positive correlation between the right upper limb and the TL. Additionally, lower limbs, trunk, subtotal body, and total body muscle content were positively correlated with TL and there was no statistically significant difference between men and women. Men have larger muscles and more fast-twitch fibers in young, healthy adults than women. Meanwhile, the decline in muscle mass, muscle strength, and body function and the increase in fat mass were more pronounced in men. During skeletal muscle development, androgens and estrogens play different roles in maintaining muscle homeostasis (71), and this leads to sex differences in skeletal muscle morphology and function. Testosterone is a pro-anabolic factor that promotes muscle protein synthesis and muscle regeneration (72). Estrogen has a protective effect on skeletal muscle by reducing inflammation (73), but currently there is no solid evidence to support the claim that estrogen affects muscle mass significantly. In addition, reports of sex differences in TL are conflicting. According to studies, estrogen protects women’s telomeres from oxidative stress, making them longer than men’s (74). However, Meyer et al. found that men had significantly longer telomeres than women (75). In conclusion, although our results require longitudinal studies to evaluate the prospective association between TL and muscle mass loss, short TL may be a risk factor for muscle loss. Meanwhile, more research is needed to advance our knowledge to the sex-specific changes affecting muscle biology.

The study has certain limitations. First, a causal relationship between TL and body composition could not be established due to the cross-sectional design of this study. In addition, despite the weighted analysis and relatively large sample size in this study, it is necessary to conduct more prospective studies involving larger sample sizes in order to determine TL’s predictive value for aging phenotypes, as well as its hypothesized role as a biomarker of aging in humans, especially bone loss. Third, part of the covariate information was collected based on self-report questionnaires, which may not accurately reflect the actual situation and introduce recall bias. Fourth, given that there is already evidence that a larger TL is associated with a healthier diet, lower alcohol intake, and more physical activity (76), all of which are associated with reduced fracture risk (77). Thus, given the differences in culture, lifestyle, and diet between different countries and regions, more research is needed to determine whether the conclusions of this study have universal applicability. In addition, our findings support the role of a healthy lifestyle in older adults as well as the link between TL and fracture risk. Fifth, it is possible that more minor fractures, such as wrist fractures, are underrecognized in the statistical data. This is because the participating population does not usually require hospitalization for these events and therefore does not know they have occurred. Therefore, our results may underestimate the effect size of fractures. Sixth, although we can account for a range of confounding factors, there is always the possibility of residual confounding. Consequently, we cannot establish causality based on these observations. Seventh, we should also use more advanced imaging methods such as high-resolution CT or MRI in the future to evaluate the relationship between TL, trabecular/cortical bone, and muscle mass/size, especially given that DXA does not distinguish between trabecular and cortical bone, and overestimates true muscle mass (78). A further problem is an inability to directly measure TL in organ-specific tissues, and it is unclear whether blood-borne TL is correlated with TL in tissues such as cartilage or bone. Finally, some unmeasured confounding variables (such as bone turnover markers), which are also considered significant factors in bone metabolism, were not evaluated in this study because they were not available in the NHANES database and the lack of adjustment for these potential factors could have biased the results.

## 5 Conclusion

Our results suggest that TL is associated with fractures and body composition at most body sites, and that this association is somewhat sex-specific. It is, however, necessary to conduct further studies in order to confirm the contrasting associations of TL with changes in the composition of the head and upper limbs, as well as the wrist fractures.

## Data availability statement

The datasets presented in this study can be found in online repositories. The names of the repository/repositories and accession number(s) can be found below: Publicly available datasets were analyzed in this study. This data can be found here: https://www.cdc.gov/nchs/nhanes/.

## Ethics statement

The studies involving humans were approved by the National Health Statistics Center’s Ethics Review Committee. The studies were conducted in accordance with the local legislation and institutional requirements. The participants provided their written informed consent to participate in this study.

## Author contributions

YG: conceptualization, methodology, material preparation, data collection, analysis and writing – original draft. HZ, FW, HX, and XL: Investigation, data collection, visualization. TH and DW: supervision, writing – review & editing. All authors contributed to the article and approved the submitted version.
