# Supplementary material for: Telomere length as a marker of changes in body composition and fractures-an analysis of data from the NHANES 2001-2002
Source: Front Immunol. 2023 Sep 8;14:1181544. doi: 10.3389/fimmu.2023.1181544 (PMC10514483; doi:10.3389/fimmu.2023.1181544)
Supplement: Supplementary file 1 [file Table_1.docx]

Supplementary Material

Telomere length as a marker of changes in body composition and fractures-an analysis of data from the NHANES 2001-2002

Youfeng Guo^1^, Haihong Zhao^1^, Feng Wang^1^, Haowei Xu^1^, Xiaowei Liu^1^, Tao Hu^1*^, Desheng Wu^1*^

*** Correspondence:** Corresponding Author: Tao Hu; dr_hutao@tongji.edu.cn, and Desheng Wu; 1300116@tongji.edu.cn

| Supplementary table S1. Types of painkillers | | |
| --- | --- | --- |
| Characteristics |  | Mean or proportion |
| Pain drugs |  |  |
|  | Aspirin | 2670(12.7) |
|  | Tylenol | 775(3.7) |
|  | Ibuprofen | 1585(7.6) |
|  | Others | 15920(76) |
| Other types of pain relievers include things like Excedrin, Vanquish, Feldene, Voltarin, Clinoril, Indocin, Naprosyn, Tolectin. | | |

| Supplementary table S2. Correlation of TL and continuous covariates by sex. | | | | |
| --- | --- | --- | --- | --- |
| Covariate | Men | | Women | |
|  | r/t | p | r/t | p |
| Age | -0.449 | < 0.001 | -0.427 | < 0.001 |
| Weight | -0.035 | < 0.001 | -0.072 | < 0.001 |
| Height | 0.095 | < 0.001 | 0.166 | < 0.001 |
| BMI | -0.103 | < 0.001 | -0.140 | < 0.001 |
| Race | 0.022 | 0.002 | 0.055 | < 0.001 |
| Education | 0.029 | < 0.001 | 0.042 | < 0.001 |
| Smoking | 0.091 | < 0.001 | 0.057 | < 0.001 |
| Physical activity level | 0.096 | < 0.001 | 0.095 | < 0.001 |
| Pain drugs | 0.089 | < 0.001 | 0.129 | < 0.001 |
| Osteoporosis | 0.033 | < 0.001 | 0.088 | < 0.001 |
| DM | 0.030 | < 0.001 | 0.120 | < 0.001 |
| TL, telomere length; DM, diabetes mellitus; BMI, body mass index. | | | | |

| Supplementary table S3. Demographic characteristics grouped by the quartile of TL. | | | | | |
| --- | --- | --- | --- | --- | --- |
| Characteristics | TL (mean or proportion) | | | | P value |
|  | Q1 | Q2 | Q3 | Q4 |  |
| Age [year], mean (SD) | 51.13(14.69) | 45.15(14.57) | 38.93(13.51) | 35.62(11.79) | < 0.001 |
| Gender, n (%) |  |  |  |  | < 0.001 |
| Male | 3555(66.2) | 2935(55.3) | 2670(49.9) | 2720(52.0) |  |
| Female | 1700(33.8) | 2290(44.7) | 2565(50.1) | 2515(48.0) |  |
| Weight [kg], mean (SD) | 84.27(20.16) | 79.48(18.46) | 78.54(17.13) | 77.39(18.37) | < 0.001 |
| Height [cm], mean (SD) | 171.88(9.59) | 170.65(10.75) | 170.88(9.28) | 171.71(9.69) | < 0.001 |
| BMI [kg/m^2^], mean (SD) | 28.40(5.91) | 27.14(5.19) | 26.79(5.05) | 26.15(5.43) | < 0.001 |
| Race, n (%) |  |  |  |  | < 0.001 |
| Mexican American | 695(3.2) | 900(5.0) | 955(6.0) | 550(3.4) |  |
| Other Hispanic | 195(6.1) | 195(4.3) | 140(3.6) | 225(4.7) |  |
| Non-Hispanic White | 3670(82.3) | 3420(83.0) | 3335(80.4) | 3515(81.6) |  |
| Non-Hispanic Black | 580(5.3) | 540(4.8) | 610(5.2) | 800(6.4) |  |
| Others | 115(3.1) | 170(2.9) | 195(5.0) | 145(3.9) |  |
| Education, n (%) |  |  |  |  | < 0.001 |
| Under high school | 895(10.5) | 650(7.7) | 810(10.7) | 580(6.6) |  |
| High school or equivalent | 1160(23.8) | 1155(23.4) | 995(19.0) | 1190(22.6) |  |
| Above high school | 3200(65.7) | 3420(68.9) | 3430(70.4) | 3465(70.8) |  |
| Smoking, n (%) |  |  |  |  | < 0.001 |
| Yes | 2640(50.8) | 2560(50.1) | 2365(44.9) | 1855(33.5) |  |
| No | 2615(49.2) | 2660(49.9) | 2870(55.1) | 3380(66.5) |  |
| Physical activity level, n (%) |  |  |  |  |  |
| Moderate | 3105(58.1) | 2860(56.1) | (48.2) | 2385(45.7) |  |
| Vigorous | 2150(41.9) | 2365(43.9) | (51.8) | 2850(54.3) |  |
| Pain drugs, n (%) |  |  |  |  | < 0.001 |
| Yes | 1820(31.8) | 1625(31.3) | 1180(22.2) | 1155(24.0) |  |
| No | 3435(68.2) | 3600(68.7) | 4055(77.8) | 4080(76.0) |  |
| SBP, mean (SD) | 123.38(17.53) | 121.46(17.30) | 117.89(13.81) | 115.21(13.61) | < 0.001 |
| DBP, mean (SD) | 73.31(10.41) | 73.23(12.81) | 71.77(9.36) | 70.79(12.80) | < 0.001 |
| Osteoporosis, n (%) |  |  |  |  | < 0.001 |
| Yes | 290(5.0) | 135(1.4) | 190(3.2) | 85(1.6) |  |
| No | 4965(95) | 5090(98.6) | 5045(96.8) | 5150(98.4) |  |
| DM, n (%) |  |  |  |  | < 0.001 |
| Yes | 535(8.5) | 260(3.3) | 285(3.9) | 110(1.0) |  |
| No | 4720(91.5) | 4965(96.7) | 4950(95.7) | 5235(99.0) |  |
| Wrist fracture, n (%) |  |  |  |  | < 0.001 |
| Yes | 460(8.1) | 670(15.2) | 800(16.7) | 495(9.9) |  |
| No | 4795(91.9) | 4555(84.8) | 4435(83.3) | 4740(90.1) |  |
| Spine fracture, n (%) |  |  |  |  | < 0.001 |
| Yes | 95(2.4) | 85(1.2) | 25(0.3) | 105(2.4) |  |
| No | 5160(97.6) | 5140(98.8) | 5210(99.7) | 5130(97.6) |  |
| BMI, body mass index; DM, diabetes mellitus; DBP, diastolic blood pressure; SBP, systolic blood pressure; TL, telomere length. TL, telomere length. Quartile TL was expressed with median[range]. Q1(0.811[≤ 0.898]); Q2(0.976[0.898-1.053]); Q3(1.131[1.053-1.220]); Q4(1.351[> 1.220]) | | | | | |

| Supplementary table S4. Body composition characteristics grouped by the quartile of TL. | | | | | |
| --- | --- | --- | --- | --- | --- |
|  | TL | | | |  |
|  | Q1 | Q2 | Q3 | Q4 | p |
| **Head** |  |  |  |  |  |
| BMC | 497.57(94.63) | 494.47(93.2) | 513.05(89.19) | 509.01(81.93) | < 0.001 |
| BMD | 2.2(0.37) | 2.19(0.36) | 2.25(0.35) | 2.24(0.32) | < 0.001 |
| Fat | 1170.2(167) | 1146.36(171.35) | 1137.21(159.98) | 1127.93(165.18) | < 0.001 |
| Lean | 3235.68(432.07) | 3178.64(456.16) | 3161(432.84) | 3141.72(444) | < 0.001 |
| **Left arm** |  |  |  |  |  |
| BMC | 201.74(54.37) | 190.91(52.4) | 191.4(48.51) | 191.16(48.76) | < 0.001 |
| BMD | 0.81(0.11) | 0.79(0.1) | 0.8(0.1) | 0.8(0.09) | < 0.001 |
| Fat | 1614.93(733.52) | 1581.46(666.17) | 1527.28(699.1) | 1432.93(661.17) | < 0.001 |
| Lean | 3211.33(1001.95) | 3082.37(1096.7) | 3047.12(1034.2) | 3021.12(1060.67) | < 0.001 |
| **Left leg** |  |  |  |  |  |
| BMC | 484.28(123.94) | 461.25(115.69) | 473.66(115.86) | 483.08(117.86) | < 0.001 |
| BMD | 1.22(0.18) | 1.2(0.15) | 1.22(0.15) | 1.24(0.15) | < 0.001 |
| Fat | 4446.55(2053.67) | 4607.56(1853.72) | 4544.94(1869.45) | 4505.57(1979) | < 0.001 |
| Lean | 8381.7(2096.75) | 8146.64(2156.62) | 8226.01(2089.94) | 8420.59(2182.25) | < 0.001 |
| **Right arm** |  |  |  |  |  |
| BMC | 211.12(56.46) | 201.06(55.11) | 202.01(51.47) | 201.78(50.57) | < 0.001 |
| BMD | 0.83(0.12) | 0.82(0.11) | 0.83(0.1) | 0.83(0.09) | < 0.001 |
| Fat | 1698.01(752.38) | 1659.95(696.39) | 1595.35(693.82) | 1502.55(678.8) | < 0.001 |
| Lean | 3341.4(999.29) | 3220.87(1130.5) | 3190.92(1060.45) | 3178.97(1083.31) | < 0.001 |
| **Right leg** |  |  |  |  |  |
| BMC | 482.84(121.1) | 463.85(114.94) | 477.86(114.5) | 483.06(114.42) | < 0.001 |
| BMD | 1.23(0.17) | 1.21(0.16) | 1.24(0.15) | 1.25(0.15) | < 0.001 |
| Fat | 4577.97(2111.12) | 4753.4(1904.01) | 4694.33(1934.32) | 4653.88(2044.95) | < 0.001 |
| Lean | 8483.4(2112.88) | 8259.23(2179.95) | 8330.45(2147.74) | 8541.17(2216.73) | < 0.001 |
| **Left rib** |  |  |  |  |  |
| BMC | 80.72(20.65) | 79.58(21.08) | 82.53(19.88) | 82.29(18.03) | < 0.001 |
| BMD | 0.67(0.1) | 0.67(0.09) | 0.68(0.09) | 0.68(0.08) | < 0.001 |
| **Right rib** |  |  |  |  |  |
| BMC | 86.7(24.85) | 85.86(23.55) | 85.64(23.03) | 86.1(21.17) | < 0.001 |
| BMD | 0.67(0.1) | 0.66(0.08) | 0.68(0.09) | 0.68(0.08) | < 0.001 |
| **Thoracic spine** |  |  |  |  |  |
| BMC | 130.73(34.2) | 126.17(27.79) | 126.24(25.49) | 124.96(26.73) | < 0.001 |
| BMD | 0.91(0.15) | 0.89(0.12) | 0.89(0.11) | 0.88(0.11) | < 0.001 |
| **Lumbar spine** |  |  |  |  |  |
| BMC | 62.58(18.11) | 59.02(16.51) | 61.53(14.65) | 60.41(13.75) | < 0.001 |
| BMD | 1.05(0.19) | 1.03(0.16) | 1.06(0.15) | 1.06(0.15) | < 0.001 |
| **Pelvis** |  |  |  |  |  |
| BMC | 259.77(84.25) | 257.26(78.32) | 278.32(82.21) | 286.48(81.64) | < 0.001 |
| BMD | 1.27(0.22) | 1.28(0.19) | 1.31(0.18) | 1.33(0.19) | < 0.001 |
| **Trunk** |  |  |  |  |  |
| BMC | 620.49(160.34) | 607.91(143.75) | 634.25(140.86) | 640.24(138.43) | < 0.001 |
| BMD | 0.94(0.15) | 0.93(0.12) | 0.96(0.12) | 0.97(0.12) | < 0.001 |
| Fat | 13939.18(5956.89) | 13417.01(5639.53) | 12411.66(5641.19) | 11442.05(5597.07) | < 0.001 |
| Lean | 26749.83(5794) | 25907.7(6064.5) | 25505.52(5449.25) | 25201.03(5685.88) | < 0.001 |
| **Subtotal** |  |  |  |  |  |
| BMC | 2000.47(494.55) | 1924.97(463.01) | 1979.17(451.83) | 1999.32(448.37) | < 0.001 |
| BMD | 1.03(0.14) | 1.01(0.12) | 1.03(0.12) | 1.04(0.11) | < 0.001 |
| Fat | 26276.63(10957.9) | 26019.36(10061.89) | 24773.56(10088.45) | 23536.98(10373.63) | < 0.001 |
| Lean | 50167.67(11595.56) | 48616.81(12311.29) | 48300.01(11472.51) | 48362.88(11842.37) | < 0.001 |
| **Total** |  |  |  |  |  |
| BMC | 2498.03(542.83) | 2419.44(505.66) | 2492.22(481.29) | 2508.34(483.56) | < 0.001 |
| BMD | 1.15(0.14) | 1.14(0.12) | 1.17(0.11) | 1.17(0.11) | < 0.001 |
| Fat | 27446.83(11020.26) | 27165.72(10114.93) | 25910.77(10135.6) | 24664.91(10444.28) | < 0.001 |
| Lean | 53403.35(11949.75) | 51795.45(12706.73) | 51461.01(11823.24) | 51504.61(12225.84) | < 0.001 |
| TL, telomere length. Quartile TL was expressed with median[range]. Q1(0.811[≤ 0.898]); Q2(0.976[0.898-1.053]); Q3(1.131[1.053-1.220]); Q4(1.351[> 1.220]). BMD, body mineral density; BMC, body mineral content. | | | | | |

| Supplementary table S5. Multiple linear regression analysis of associations between TL and body composition measurements (merged ribs and limbs of the left and right body). | | | | |
| --- | --- | --- | --- | --- |
|  | Model1 | Model2 | Model3 | Model4 |
| **Limbs** |  |  |  |  |
| BMC | 0.025[13.229, 44.735] | 0.043[39.618, 60.949] | 0.037[32.444, 53.750] | 0.035[30.359, 51.763] |
| BMD | 0.046[0.056, 0.102] | 0.016[0.012, 0.044] | 0.012[0.004, 0.036] | 0.010[0.004, 0.033] |
| Fat | -0.080[-1683.092, -1195.013] | 0.003[-62.232, 174.405] | -0.001[-128.032, 109.011] | 0.001[-108.967, 129.283] |
| Lean | 0.011[-55.136, 551.075] | 0.055[1085.484, 1373.928] | 0.049[945.691, 1232.678] | 0.046[885.933, 1173.875] |
| **Ribs** |  |  |  |  |
| BMC | 0.009[-0.644, 3.187] | 0.027[2.378, 5.188] | 0.021[1.631, 4.446] | 0.017[0.997, 3.824] |
| BMD | 0.063[0.028, 0.044] | 0.016[0.002, 0.016] | 0.014[0.001, 0.015] | 0.011[-0.001, 0.013] |
| BMC, bone mineral content; BMD, bone mineral density.  Model 1: Unadjusted model.  Model 2: age, gender, race, and BMI were adjusted.  Model 3: age, gender, race, BMI, education and smoking were adjusted.  Model 4: age, gender, race, BMI, education, smoking, osteoporosis, DM history, pain drugs intake history, and physical activity level were adjusted. | | | | |

| Supplementary table S6. Sex-specific multiple linear regression analysis of associations between TL and body composition measurements. | | | |
| --- | --- | --- | --- |
|  | Male | Female | p-interaction |
| **Head** |  |  |  |
| BMC | -0.047*** | 0.024* | *** |
| BMD | -0.029** | 0.031** | *** |
| Fat | -0.039*** | -0.005 | 0.077 |
| Lean | -0.035*** | -0.002 | 0.163 |
| **Left arm** |  |  |  |
| BMC | 0.005 | 0.003 | *** |
| BMD | -0.016 | -0.018 | *** |
| Fat | -0.010* | -0.008 | *** |
| Lean | 0.008 | -0.016* | *** |
| **Left leg** |  |  |  |
| BMC | 0.105*** | 0.055*** | 0.154 |
| BMD | 0.067*** | 0.076*** | *** |
| Fat | -0.002 | 0.006 | ** |
| Lean | 0.109*** | 0.062*** | *** |
| **Right arm** |  |  |  |
| BMC | -0.010 | -0.001 | *** |
| BMD | -0.061*** | -0.040*** | *** |
| Fat | -0.008 | -0.007 | *** |
| Lean | 0.006 | -0.007 | *** |
| **Right leg** |  |  |  |
| BMC | 0.086*** | 0.039* | 0.260 |
| BMD | 0.038*** | 0.016 | *** |
| Fat | 0.006 | 0.012 | ** |
| Lean | 0.121*** | 0.066*** | *** |
| **Left rib** |  |  |  |
| BMC | -0.004 | 0.013 | *** |
| BMD | 0.018 | -0.035*** | *** |
| **Right rib** |  |  |  |
| BMC | 0.009 | 0.073*** | *** |
| BMD | 0.065*** | -0.014 | ** |
| **Thoracic spine** |  |  |  |
| BMC | 0.019* | 0.067*** | *** |
| BMD | -0.013 | -0.013 | *** |
| **Lumbar spine** |  |  |  |
| BMC | -0.002 | -0.088*** | *** |
| BMD | 0.042*** | -0.036*** | *** |
| Pelvis |  |  |  |
| BMC | 0.106*** | 0.041*** | *** |
| BMD | 0.006 | 0.006 | 0.266 |
| **Trunk** |  |  |  |
| BMC | 0.067*** | 0.042*** | 0.514 |
| BMD | 0.044*** | -0.019 | ** |
| Fat | -0.053*** | -0.0004 | 0.720 |
| Lean | 0.054*** | 0.043*** | 0.308 |
| **Subtotal** |  |  |  |
| BMC | 0.072*** | 0.039*** | 0.618 |
| BMD | 0.043*** | 0.009 | *** |
| Fat | -0.032*** | 0.002 | 0.160 |
| Lean | 0.073*** | 0.047*** | *** |
| **Total** |  |  |  |
| BMC | 0.056*** | 0.040** | 0.074 |
| BMD | 0.020* | 0.002 | *** |
| Fat | -0.032*** | 291.709 | 0.170 |
| Lean | 0.070*** | 0.046*** | *** |
| Age, race, BMI, education, smoking, osteoporosis, DM history, pain drugs intake history, and physical activity level were adjusted. BMI, body mass index; BMD, body mineral density; BMC, body mineral content. ∗ represents p ＜ 0.05, ** represents p < 0.01, *** represents p < 0.001. | | | |

| Supplementary table S7. Age-specific multiple linear regression analysis of associations between TL and body composition measurements. | | | | | | | | |
| --- | --- | --- | --- | --- | --- | --- | --- | --- |
|  | 20 ≤ age < 30 | 30 ≤ age < 40 | 40 ≤ age < 50 | 50 ≤ age < 60 | 60 ≤ age < 70 | 70 ≤ age < 80 | 80 ≤ age ≤ 85 | p-interaction |
| **Head** |  |  |  |  |  |  |  |  |
| BMC | 0.001 | -0.024 | -0.101 | 0.103*** | -0.048* | 0.034 | -0.055 | *** |
| BMD | 0.024 | -0.010 | -0.071*** | 0.145*** | -0.082*** | 0.041 | -0.049 | *** |
| Fat | -0.028** | -0036*** | -0.011 | -0.049*** | 0.021 | 0.066*** | 0.042 | *** |
| Lean | -0.024** | -0.029** | -0.023** | -0.037*** | 0.031* | 0.060*** | 0.041* | *** |
| **Left arm** |  |  |  |  |  |  |  |  |
| BMC | 0.058*** | -0.071*** | 0.013 | 0.006 | -0.036** | 0.038** | 0.022 | *** |
| BMD | 0.044*** | -0.095*** | -0.024* | 0.074*** | -0.074*** | 0.009 | -0.017 | *** |
| Fat | -0.010 | -0.041*** | 0.027*** | -0.055*** | 0.022* | 0.047*** | 0.082*** | *** |
| Lean | 0.031*** | -0.037*** | 0.017 | -0.022** | -0.045*** | 0.090*** | -0.046* | *** |
| **Left leg** |  |  |  |  |  |  |  |  |
| BMC | 0.142*** | -0.057*** | 0.078*** | 0.002 | -0.044*** | 0.078*** | 0.027 | * |
| BMD | 0.140*** | -0.026*** | 0.021** | -0.046* | -0.016 | 0.018* | 0.049* | ** |
| Fat | 0.018** | -0.019* | -0.043*** | -0.016 | 0.083*** | -0.069*** | 0.048* | *** |
| Lean | 0.070*** | 0.056*** | 0.117*** | -0.023* | -0.023** | 0.086*** | -0.054** | *** |
| **Right arm** |  |  |  |  |  |  |  |  |
| BMC | 0.039*** | -0.085*** | 0.015 | 0.031** | -0.073*** | 0.042** | 0.005 | *** |
| BMD | 0.004 | -0.128*** | -0.026* | 0.070*** | -0.106*** | 0.015 | -0.014 | *** |
| Fat | -0.019*** | -0.026*** | 0.021** | -0.046*** | -0.016 | 0.017 | 0.043* | *** |
| Lean | 0.012 | -0.046*** | 0.041*** | 0.002 | -0.066*** | 0.082*** | -0.076*** | *** |
| **Right leg** |  |  |  |  |  |  |  |  |
| BMC | 0.116*** | -0.055*** | 0.066*** | -0.001 | -0.028* | 0.051** | 0.040 | 0.382 |
| BMD | 0.085*** | -0.075*** | 0.025* | 0.005 | -0.058*** | 0.011 | 0.034 | * |
| Fat | 0.022*** | -0.013 | -0.038*** | -0.004 | 0.096*** | -0.064*** | 0.051** | *** |
| Lean | 0.077*** | 0.055*** | 0.124*** | -0.003 | -0.009 | 0.066*** | -0.026 | *** |
| **Left rib** |  |  |  |  |  |  |  |  |
| BMC | 0.026* | -0.084*** | 0.092*** | 0.060*** | -0.107*** | 0.017 | 0.057* | ** |
| BMD | 0.043*** | -0.100*** | -0.007 | 0.108*** | -0.098*** | 0.050* | -0.078** | 0.078 |
| **Right rib** |  |  |  |  |  |  |  |  |
| BMC | 0.101*** | -0.045*** | 0.022* | 0.069*** | 0.015 | 0.164*** | 0.0001 | *** |
| BMD | 0.035** | -0.045** | 0.018 | 0.152*** | -0.060*** | 0.108*** | 0.014 | 0.064 |
| **Thoracic spine** |  |  |  |  |  |  |  |  |
| BMC | 0.089*** | -0.110*** | 0.046*** | 0.090*** | -0.065*** | 0.086*** | -0.058* | *** |
| BMD | 0.032* | -0.117*** | -0.014 | 0.071*** | -0.081*** | 0.050** | 0.024 | *** |
| **Lumbar spine** |  |  |  |  |  |  |  |  |
| BMC | 0.022 | -0.165*** | 0.006 | -0.031 | -0.153*** | 0.086*** | -0.004 | * |
| BMD | 0.095*** | -0.188*** | 0.082*** | 0.074*** | -0.189*** | 0.040 | 0.016 | *** |
| **Pelvis** |  |  |  |  |  |  |  |  |
| BMC | 0.129*** | -0.041 | 0.108*** | 0.032* | -0.068*** | 0.083*** | -0.004 | *** |
| BMD | 0.045*** | -0.118*** | 0.105*** | 0.006 | -0.116*** | 0.043* | 0.021 | *** |
| **Trunk** |  |  |  |  |  |  |  |  |
| BMC | 0.114*** | -0.081*** | 0.085*** | 0.052*** | -0.078*** | 0.100*** | -0.009 | 0.907 |
| BMD | 0.065*** | -0.117*** | 0.059*** | 0.062*** | -0.116*** | 0.062** | -0.002 | 0.287 |
| Fat | -0.017** | -0.051*** | -0.016*** | -0.039*** | -0.031*** | 0.114*** | 0.046* | *** |
| Lean | 0.050*** | 0.009 | 0.050*** | 0.021** | -0.033*** | 0.086*** | -0.033 | *** |
| **Subtotal** |  |  |  |  |  |  |  |  |
| BMC | 0.112*** | -0.071*** | 0.066*** | 0.022 | -0.055*** | 0.072*** | 0.017 | 0.865 |
| BMD | 0.091*** | -0.102*** | 0.044*** | 0.048** | -0.100*** | 0.038* | 0.009 | 0.842 |
| Fat | -0.003*** | -0.039*** | -0.021*** | -0.033*** | 0.021*** | 0.041** | 0.054** | *** |
| Lean | 0.054*** | 0.017* | 0.073*** | 0.004 | -0.032*** | 0.085*** | -0.041* | *** |
| **Total** |  |  |  |  |  |  |  |  |
| BMC | 0.105*** | -0.071*** | 0.047*** | 0.039** | -0.061*** | 0.072*** | 0.008 | * |
| BMD | 0.083*** | -0.102*** | 0.003 | 0.081*** | -0.112*** | 0.039 | -0.013 | *** |
| Fat | -0.003 | -0.039*** | -0.021*** | -0.033*** | 0.021** | 0.042*** | 0.055** | *** |
| Lean | 0.052*** | 0.015* | 0.069*** | 0.003 | -0.030*** | 0.085*** | -0.037* | *** |
| Gender, race, BMI, education, smoking, osteoporosis, DM history, pain drugs intake history, and physical activity level were adjusted. BMI, body mass index; BMD, body mineral density; BMC, body mineral content. ∗ represents p ＜ 0.05, ** represents p < 0.01, *** represents p < 0.001. | | | | | | | | |

| Supplementary table S8. BMI-specific multiple linear regression analysis of associations between TL and body composition measurements. | | | | |
| --- | --- | --- | --- | --- |
|  | BMI < 25 kg/m2 | 25 ≤ BMI < 30 kg/m2 | BMI ≥ 30 kg/m2 | p-interaction |
| **Head** |  |  |  |  |
| BMC | -0.050 | 0.012 | -0.052*** | * |
| BMD | -0.002 | 0.012 | -0.063*** | 0.255 |
| Fat | -0.050*** | 0.004 | -0.024* | ** |
| Lean | -0.050*** | 0.010 | -0.015 | *** |
| **Left arm** |  |  |  |  |
| BMC | -0.010 | 0.061*** | -0.111*** | 0.053 |
| BMD | -0.024 | 0.030*** | -0.073*** | * |
| Fat | -0.005 | -0.050** | -0.024 | * |
| Lean | -0.003 | 0.060*** | -0.076*** | 0.397 |
| **Left leg** |  |  |  |  |
| BMC | 0.047*** | 0.161*** | -0.090*** | 0.229 |
| BMD | 0.032** | 0.144*** | -0.071*** | 0.755 |
| Fat | 0.001 | -0.042*** | 0.021 | *** |
| Lean | 0.086*** | 0.125*** | -0.005 | * |
| **Right arm** |  |  |  |  |
| BMC | 0.020** | 0.053*** | -0.101*** | ** |
| BMD | 0.044*** | 0.006 | -0.097*** | 0.365 |
| Fat | -0.019 | -0.049*** | -0.007 | * |
| Lean | -0.006 | 0.062*** | -0.064*** | * |
| **Right leg** |  |  |  |  |
| BMC | 0.030** | 0.141*** | -0.074*** | * |
| BMD | -0.022* | 0.104*** | -0.048*** | * |
| Fat | 0.009 | -0.027** | 0.024* | *** |
| Lean | 0.080*** | 0.134*** | 0.014 | ** |
| **Left rib** |  |  |  |  |
| BMC | 0.020* | 0.083*** | -0.110*** | 0.496 |
| BMD | -0.005 | 0.072*** | -0.145*** | 0.509 |
| **Right rib** |  |  |  |  |
| BMC | 0.108*** | 0.065*** | -0.072*** | *** |
| BMD | 0.005 | 0.114*** | -0.075*** | 0.993 |
| **Thoracic spine** |  |  |  |  |
| BMC | 0.127*** | 0.042*** | -0.121*** | *** |
| BMD | 0.005 | 0.021 | -0.146*** | *** |
| **Lumbar spine** |  |  |  |  |
| BMC | 0.042*** | 0.037** | -0.201*** | *** |
| BMD | -0.0002 | 0.100*** | -0.183*** | *** |
| **Pelvis** |  |  |  |  |
| BMC | 0.054*** | 0.173*** | -0.098*** | *** |
| BMD | 0.026* | 0.072*** | -0.120*** | * |
| **Trunk** |  |  |  |  |
| BMC | 0.067*** | 0.136*** | -0.126*** | *** |
| BMD | -0.007 | 0.105*** | -0.142*** | *** |
| Fat | -0.007 | -0.142*** | -0.021 | * |
| Lean | 0.059*** | 0.109*** | -0.084*** | ** |
| **Subtotal** |  |  |  |  |
| BMC | 0.037*** | 0.132*** | -0.105*** | 0.597 |
| BMD | 0.001 | 0.108*** | -0.098*** | 0.45 |
| Fat | -0.003 | -0.098*** | -0.003 | ** |
| Lean | 0.058*** | 0.111*** | -0.053*** | 0.996 |
| **Total** |  |  |  |  |
| BMC | 0.025** | 0.125*** | -0.106*** | 0.907 |
| BMD | -0.020 | 0.099*** | -0.099*** | 0.770 |
| Fat | -0.004 | -0.098*** | -0.003 | *** |
| Lean | 0.054*** | 0.108*** | -0.052*** | 0.857 |
| Age, gender, race, education, smoking, osteoporosis, DM history, pain drugs intake history, and physical activity level were adjusted. BMD, body mineral density; BMC, body mineral content. ∗ represents p ＜ 0.05, ** represents p < 0.01, *** represents p < 0.001. | | | | |

| Supplementary table S9. Race-specific multiple linear regression analysis of associations between TL and body composition measurements. | | | | | |
| --- | --- | --- | --- | --- | --- |
|  | Mexican American | Other Hispanic | Non-Hispanic White | Non-Hispanic Black | p-interaction |
| **Head** |  |  |  |  |  |
| BMC | 0.198*** | 0.220*** | -0.054*** | -0.086*** | *** |
| BMD | 0.181*** | 0.279*** | -0.035*** | -0.083*** | 0.177 |
| Fat | -0.011 | 0.033 | -0.039*** | -0.026 | 0.401 |
| Lean | -0.010 | 0.037 | -0.035*** | -0.018 | 0.736 |
| **Left arm** |  |  |  |  |  |
| BMC | 0.032* | 0.080** | -0.016** | -0.075*** | ** |
| BMD | 0.087*** | 0.262*** | -0.038*** | -0.099*** | *** |
| Fat | -0.047*** | -0.065*** | -0.006 | 0.016* | *** |
| Lean | 0.024** | -0.039** | -0.005 | -0.066*** | 0.564 |
| **Left leg** |  |  |  |  |  |
| BMC | 0.045** | -0.061* | 0.060*** | -0.041** | 0.205 |
| BMD | 0.087*** | -0.041 | 0.054*** | -0.063*** | 0.063 |
| Fat | -0.086*** | 0.073*** | -0.010* | 0.033** | ** |
| Lean | 0.091*** | -0.104*** | 0.071*** | -0.007 | 0.495 |
| **Right arm** |  |  |  |  |  |
| BMC | 0.028* | 0.014 | -0.019*** | -0.065*** | ** |
| BMD | 0.079*** | 0.088*** | -0.059*** | -0.083*** | *** |
| Fat | -0.058*** | -0.039* | -0.009* | 0.021** | *** |
| Lean | 0.030** | -0.045** | -0.004 | -0.035*** | 0.945 |
| **Right leg** |  |  |  |  |  |
| BMC | 0.050** | -0.047 | 0.047*** | -0.042** | 0.111 |
| BMD | 0.062*** | 0.005 | 0.013* | -0.057*** | ** |
| Fat | -0.072*** | 0.077*** | -0.001 | 0.024* | ** |
| Lean | 0.083*** | -0.077*** | 0.078*** | -0.009 | 0.901 |
| **Left rib** |  |  |  |  |  |
| BMC | 0.090*** | -0.008 | -0.004 | -0.096*** | *** |
| BMD | 0.068*** | 0.057 | -0.007 | -0.160*** | *** |
| **Right rib** |  |  |  |  |  |
| BMC | 0.029* | 0.125*** | 0.031*** | -0.076*** | 0.370 |
| BMD | 0.057** | 0.046 | 0.032*** | -0.160*** | ** |
| **Thoracic spine** |  |  |  |  |  |
| BMC | 0.064*** | 0.065* | 0.020** | -0.164*** | *** |
| BMD | 0.117*** | 0.137*** | -0.033*** | -0.155*** | *** |
| **Lumbar spine** |  |  |  |  |  |
| BMC | 0.126*** | -0.061 | -0.049*** | -0.227*** | *** |
| BMD | 0.068*** | 0.118*** | -0.002 | -0.189*** | *** |
| **Pelvis** |  |  |  |  |  |
| BMC | 0.104*** | -0.003 | 0.074*** | -0.073*** | *** |
| BMD | 0.141*** | 0.148*** | -0.002 | -0.038* | * |
| **Trunk** |  |  |  |  |  |
| BMC | 0.102*** | 0.029 | 0.046*** | -0.122*** | *** |
| BMD | 0.127*** | 0.118*** | 0.008 | -0.120*** | *** |
| Fat | -0.050*** | -0.001 | -0.034*** | 0.006 | *** |
| Lean | 0.039*** | -0.003 | 0.023*** | -0.072*** | * |
| **Subtotal** |  |  |  |  |  |
| BMC | 0.065*** | -0.009 | 0.037*** | -0.076*** | ** |
| BMD | 0.107*** | 0.066** | 0.012 | -0.090*** | *** |
| Fat | -0.063*** | 0.019 | -0.022*** | 0.016** | *** |
| Lean | 0.055*** | -0.041** | 0.037*** | -0.046*** | 0.162 |
| **Total** |  |  |  |  |  |
| BMC | 0.102*** | 0.032 | 0.026*** | -0.088*** | *** |
| BMD | 0.168*** | 0.176*** | -0.009 | -0.105*** | *** |
| Fat | -0.063*** | 0.019 | -0.023*** | 0.016** | *** |
| Lean | 0.053*** | -0.038** | 0.035*** | -0.045*** | 0.179 |
| Age, gender, BMI, education, smoking, osteoporosis, DM history, pain drugs intake history, and physical activity level were adjusted. BMI, body mass index; BMD, body mineral density; BMC, body mineral content. ∗ represents p ＜ 0.05, ** represents p < 0.01, *** represents p < 0.001. | | | | | |

| Supplementary table S10. Sex-specific logistic regressions for association between fracture and TL. | | | | |
| --- | --- | --- | --- | --- |
| Outcome | Men | | Women | |
|  | OR (95%CI) | P | OR (95%CI) | P |
| Wrist fracture | 0.872(0.706-1.076) | 0.201 | 1.243(0.889-1.736) | 0.203 |
| Spine fracture | 0.307(0.190-0.496) | < 0.001 | 1.282(0.494-3.327) | 0.609 |
| TL, telomere length. | | | | |

| Supplementary table S11. Sex-specific trend analysis of logistic regressions for association between fracture and TL. | | | | | |
| --- | --- | --- | --- | --- | --- |
| Category | Outcome | Q1 | Q2 | Q3 | Q4 |
|  |  | OR (95%CI) | OR (95%CI) | OR (95%CI) | OR (95%CI) |
| Men | Wrist fracture | 1.205(1.008-1.441) | 0.793(0.675-0.932) | 0.683(0.585-0.798) | Reference |
|  |  | p_trend_ < 0.001 | | | |
|  | Spine fracture | 2.640(1.770-3.937) | 2.520(1.714-3.705) | 6.380(3.668-11.096) | Reference |
|  |  | p_trend_ < 0.001 | | | |
| Women | Wrist fracture | 0.558(0.430-0.725) | 0.607(0.483-0.763) | 0.333(0.269-0.411) | Reference |
|  |  | p_trend_ < 0.001 | | | |
|  | Spine fracture | 0.790(0.411-1.516) | 0.874(0.459-1.667) | 2.646(1.159-6.037) | Reference |
|  |  | p_trend_ = 0.033 | | | |
| TL, telomere length. Quartile TL was expressed with median[range]. Q1(0.811[≤ 0.898]); Q2(0.976[0.898-1.053]); Q3(1.131[1.053-1.220]); Q4(1.351[> 1.220]) | | | | | |

| Supplementary table S12. Age or BMI-specific logistic regressions for association between fracture and TL. | | | |
| --- | --- | --- | --- |
|  |  | Wrist fracture | Spine fracture |
|  |  | OR [95%CI] | OR [95%CI] |
| Age |  |  |  |
|  | 20 ≤ age < 30 | 2.016(1.465, 2.772) | 3.030(0.777, 11.815) |
|  | 30 ≤ age < 40 | 2.513(1.537, 4.107) | 4.357(1.068, 17.779) |
|  | 40 ≤ age < 50 | 0.745(0.508, 1.092) | 0.041(0.019, 0.091) |
|  | 50 ≤ age < 60 | 0.182(0.107, 0.310) | 1.583(0.279, 8.973) |
|  | 60 ≤ age < 70 | 0.097(0.049, 0.195) | 0.042(0.007, 0.272) |
|  | 70 ≤ age < 80 | 15.225(5.282, 43.880) | 0.066(0.012, 0.377) |
|  | 80 ≤ age ≤ 85 | 5.196(0.429, 62.963) | 0.836(0.053, 13.321) |
| BMI |  |  |  |
|  | BMI < 25 kg/m2 | 1.699(1.271, 2.271) | 0.102(0.046, 0.224) |
|  | 25 ≤ BMI < 30 kg/m2 | 1.647(1.201, 2.260) | 0.040(0.021, 0.076) |
|  | BMI ≥ 30 kg/m2 | 0.122 (0.084, 0.178) | 1.017(1.001, 1.033) |
| TL, telomere length; BMI, body mass index; DM, diabetes mellitus; BMD, bone mineral density. | | | |

| Supplementary Table S13. BMD at different skeletal sites among different age groups | | | | | | | |
| --- | --- | --- | --- | --- | --- | --- | --- |
|  | 20 ≤ age < 30 | 30 ≤ age < 40 | 40 ≤ age < 50 | 50 ≤ age < 60 | 60 ≤ age < 70 | 70 ≤ age < 80 | 80 ≤ age ≤ 85 |
| **Head** |  |  |  |  |  |  |  |
| BMC | 497.68(79.81) | 524.73(85.42) | 518.86(80.07) | 505.49(97.37) | 491.88(99.16) | 468.30(99.37) | 446.97(91.36) |
| BMD | 2.16(0.30) | 2.29(0.33) | 2.28(0.32) | 2.24(0.38) | 2.21(0.40) | 2.12(0.37) | 2.04(0.36) |
| Fat | 1130.97(157.02) | 1140.23(171.67) | 1162.99(168.93) | 1173.49(174.78) | 1141.40(167.85) | 1134.82(148.32) | 1081.65(152.74) |
| Lean | 3157.65(424.31) | 3173.20(468.05) | 3220.41(439.45) | 3243.74(454.89) | 3156.15(443.20) | 3138.89(395.39) | 3006.26(411.36) |
| **Left arm** |  |  |  |  |  |  |  |
| BMC | 189.73(47.41) | 195.64(49.96) | 202.76(48.89) | 199.44(48.11) | 186.41(56.61) | 189.03(56.60) | 168.38(60.49) |
| BMD | 0.81(0.09) | 0.82(0.10) | 0.82(0.09) | 0.80(0.10) | 0.77(0.11) | 0.77(0.12) | 0.72(0.13) |
| Fat | 1345.64(673.14) | 1465.62(694.94) | 1592.17(739.20) | 1716.67(722.18) | 1679.54(654.09) | 1626.53(523.97) | 1438.40(506.14) |
| Lean | 3062.47(1071.12) | 3151.16(1102.37) | 3306.69(1077.08) | 3187.01(1015.42) | 2865.54(995.53) | 2896.31(828.27) | 2443.56(754.38) |
| **Left leg** |  |  |  |  |  |  |  |
| BMC | 488.39(118.30) | 481.13(120.73) | 490.34(109.01) | 479.64(112.78) | 447.72(114.66) | 448.45(123.75) | 414.62(144.36) |
| BMD | 1.25(0.15) | 1.25(0.15) | 1.24(0.13) | 1.21(0.15) | 1.17(0.17) | 1.15(0.17) | 1.11(0.21) |
| Fat | 4425.71(1984.72) | 4376.57(1918.92) | 4660.37(2059.34) | 4834.46(2027.02) | 4652.74(1902.85) | 4332.18(1571.45) | 3917.01(1140.25) |
| Lean | 8410.48(2163.63) | 8418.52(2263.29) | 8719.04(2107.00) | 8512.72(2098.05) | 7711.23(1986.28) | 7678.65(1675.00) | 6784.28(1549.65) |
| **Right arm** |  |  |  |  |  |  |  |
| BMC | 201.60(50.43) | 207.35(53.59) | 213.59(51.33) | 208.22(50.73) | 194.76(55.75) | 196.45(57.50) | 174.33(62.23) |
| BMD | 0.84(0.09) | 0.85(0.10) | 0.85(0.09) | 0.83(0.10) | 0.79(0.11) | 0.79(0.12) | 0.73(0.13) |
| Fat | 1420.22(697.03) | 1526.58(710.85) | 1670.38(751.06) | 1793.23(715.55) | 1765.84(699.68) | 1713.27(534.66) | 1497.46(479.54) |
| Lean | 3221.40(1095.06) | 3308.47(1148.44) | 3459.25(1077.49) | 3316.44(1023.15) | 2986.23(1012.08) | 2998.18(827.32) | 2563.96(761.19) |
| **Right leg** |  |  |  |  |  |  |  |
| BMC | 490.41(115.84) | 483.88(116.80) | 491.98(106.47) | 480.48(111.97) | 446.71(112.30) | 449.27(121.49) | 413.06(143.67) |
| BMD | 1.27(0.15) | 1.26(0.15) | 1.25(0.13) | 1.23(0.15) | 1.17(0.16) | 1.16(0.18) | 1.12(0.21) |
| Fat | 4570.57(2049.06) | 4524.70(1974.10) | 4808.83(2098.50) | 4996.37(2113.66) | 4778.08(1965.91) | 4471.23(1631.96) | 3997.80(1172.23) |
| Lean | 8515.57(2198.44) | 8561.82(2310.42) | 8809.51(2132.33) | 8630.32(2156.74) | 7781.42(2003.05) | 7830.52(1663.03) | 6860.27(1546.81) |
| **Left rib** |  |  |  |  |  |  |  |
| BMC | 85.59(18.07) | 84.02(21.25) | 84.10(17.97) | 82.02(19.69) | 72.92(19.12) | 73.74(19.63) | 64.49(19.22) |
| BMD | 0.70(0.07) | 0.69(0.09) | 0.69(0.08) | 0.67(0.08) | 0.64(0.09) | 0.64(0.10) | 0.60(0.10) |
| **Right rib** |  |  |  |  |  |  |  |
| BMC | 86.93(21.20) | 87.17(23.79) | 89.60(23.65) | 88.00(22.98) | 81.98(23.17) | 81.07(21.22) | 70.91(24.74) |
| BMD | 0.70(0.08) | 0.68(0.09) | 0.68(0.08) | 0.66(0.08) | 0.65(0.09) | 0.66(0.10) | 0.60(0.10) |
| **Thoracic spine** |  |  |  |  |  |  |  |
| BMC | 121.56(24.14) | 125.09(23.70) | 133.51(29.14) | 134.28(32.26) | 125.86(30.42) | 125.69(31.03) | 110.11(35.64) |
| BMD | 0.88(0.09) | 0.89(0.10) | 0.91(0.12) | 0.91(0.13) | 0.89(0.14) | 0.89(0.16) | 0.84(0.17) |
| **Lumbar spine** |  |  |  |  |  |  |  |
| BMC | 61.60(13.00) | 61.77(13.67) | 62.69(15.07) | 59.46(15.81) | 58.08(18.11) | 61.29(19.81) | 55.27(25.15) |
| BMD | 1.09(0.14) | 1.07(0.15) | 1.06(0.15) | 1.03(0.17) | 1.02(0.18) | 1.03(0.20) | 0.99(0.24) |
| **Pelvis** |  |  |  |  |  |  |  |
| BMC | 307.24(87.06) | 286.18(74.21) | 275.98(72.80) | 257.31(78.94) | 233.20(69.64) | 226.76(73.81) | 200.18(73.63) |
| BMD | 1.35(0.19) | 1.35(0.18) | 1.34(0.18) | 1.28(0.20) | 1.21(0.18) | 1.17(0.18) | 1.07(0.20) |
| **Trunk** |  |  |  |  |  |  |  |
| BMC | 662.92(142.48) | 644.23(133.24) | 645.88(134.62) | 621.06(149.22) | 572.03(141.32) | 568.55(143.74) | 500.96(164.74) |
| BMD | 0.99(0.11) | 0.98(0.11) | 0.97(0.11) | 0.94(0.13) | 0.90(0.13) | 0.90(0.14) | 0.83(0.15) |
| Fat | 10561.71(5577.27) | 11744.41(5630.26) | 13553.34(6103.83) | 14717.31(5817.58) | 14162.94(5225.26) | 14413.14(4436.30) | 11996.06(4321.79) |
| Lean | 25006.61(5544.00) | 25655.38(5895.34) | 26968.18(5799.04) | 27042.78(5951.61) | 25064.75(5755.16) | 25726.58(5157.64) | 23200.52(4845.00) |
| **Subtotal** |  |  |  |  |  |  |  |
| BMC | 2033.05(456.27) | 2012.24(453.73) | 2044.56(427.37) | 1988.85(451.96) | 1847.62(463.37) | 1851.74(484.77) | 1671.35(560.52) |
| BMD | 1.06(0.11) | 1.05(0.11) | 1.05(0.11) | 1.02(0.12) | 0.98(0.13) | 0.97(0.14) | 0.92(0.16) |
| Fat | 22323.86(10572.07) | 23637.87(10337.86) | 26285.09(11052.20) | 28058.05(10543.40) | 27039.14(9615.16) | 26556.36(7870.86) | 22846.74(6982.87) |
| Lean | 48216.52(11724.60) | 49095.35(12350.56) | 51262.67(11830.41) | 50689.28(11920.53) | 46409.17(11434.77) | 47130.23(9812.35) | 41852.59(9247.98) |
| **Total** |  |  |  |  |  |  |  |
| BMC | 2530.73(485.80) | 2536.97(487.39) | 2563.42(456.06) | 2494.34(496.27) | 2339.50(501.13) | 2320.04(540.77) | 2118.32(618.28) |
| BMD | 1.18(0.10) | 1.19(0.11) | 1.18(0.10) | 1.15(0.12) | 1.11(0.13) | 1.09(0.14) | 1.05(0.15) |
| Fat | 23454.83(10637.86) | 24778.11(10405.50) | 27448.07(11117.09) | 29231.54(10594.12) | 28180.54(9658.49) | 27691.18(7890.82) | 23928.40(7042.52) |
| Lean | 51374.17(12082.87) | 52268.54(12753.00) | 54483.08(12195.88) | 53933.01(12310.82) | 49565.32(11798.88) | 50269.13(10135.68) | 44858.84(9589.50) |
| BMI, body mass index; BMD, body mineral density; BMC, body mineral content. | | | | | | | |

| Supplementary Table S14. BMD at different skeletal sites among different age or BMI groups | | | |
| --- | --- | --- | --- |
|  | BMI | | |
|  | Normal# | Overweight | Obese |
| **Head** |  |  |  |
| BMC | 496.37(80.77) | 510.25(95.06) | 503.64(94.55) |
| BMD | 2.19(0.31) | 2.22(0.37) | 2.26(0.37) |
| Fat | 1045.60(119.23) | 1170.19(134.62) | 1249.53(188.13) |
| Lean | 2931.95(340.47) | 3257.43(375.43) | 3412.28(490.83) |
| **Left arm** |  |  |  |
| BMC | 174.62(46.68) | 203.05(49.82) | 207.18(51.55) |
| BMD | 0.78(0.09) | 0.81(0.10) | 0.82(0.10) |
| Fat | 1028.51(315.13) | 1493.69(348.38) | 2326.40(750.96) |
| Lean | 2584.11(863.44) | 3245.39(969.87) | 3575.26(1106.74) |
| **Left leg** |  |  |  |
| BMC | 437.92(106.40) | 492.98(117.74) | 502.91(123.19) |
| BMD | 1.18(0.15) | 1.24(0.16) | 1.25(0.16) |
| Fat | 3410.97(1099.10) | 4294.42(1283.84) | 6440.72(2241.69) |
| Lean | 7119.10(1680.57) | 8461.48(1807.98) | 9701.53(2221.72) |
| **Right arm** |  |  |  |
| BMC | 184.85(48.73) | 213.37(51.85) | 217.13(55.10) |
| BMD | 0.80(0.10) | 0.84(0.10) | 0.85(0.11) |
| Fat | 1085.08(320.23) | 1560.05(336.56) | 2439.48(753.44) |
| Lean | 2727.55(886.23) | 3386.33(985.20) | 3718.93(1135.35) |
| **Right leg** |  |  |  |
| BMC | 438.89(104.21) | 493.60(115.03) | 505.79(121.04) |
| BMD | 1.19(0.15) | 1.25(0.16) | 1.26(0.16) |
| Fat | 3509.61(1129.83) | 4439.15(1335.10) | 6646.50(2293.82) |
| Lean | 7230.47(1723.86) | 8570.81(1846.23) | 9809.88(2254.37) |
| **Left rib** |  |  |  |
| BMC | 72.58(16.40) | 82.05(17.37) | 92.39(22.28) |
| BMD | 0.66(0.09) | 0.68(0.09) | 0.69(0.09) |
| **Right rib** |  |  |  |
| BMC | 72.08(16.74) | 87.69(18.09) | 103.40(25.16) |
| BMD | 0.65(0.09) | 0.68(0.08) | 0.70(0.09) |
| **Thoracic spine** |  |  |  |
| BMC | 113.69(22.04) | 128.64(27.11) | 143.44(30.63) |
| BMD | 0.85(0.11) | 0.89(0.11) | 0.95(0.13) |
| **Lumbar spine** |  |  |  |
| BMC | 59.95(14.54) | 61.82(16.99) | 60.84(15.98) |
| BMD | 1.04(0.16) | 1.05(0.17) | 1.08(0.16) |
| **Pelvis** |  |  |  |
| BMC | 259.68(77.73) | 272.51(83.79) | 282.60(85.36) |
| BMD | 1.22(0.17) | 1.31(0.19) | 1.38(0.21) |
| **Trunk** |  |  |  |
| BMC | 577.98(130.48) | 632.71(140.96) | 682.68(153.98) |
| BMD | 0.92(0.12) | 0.96(0.13) | 0.98(0.13) |
| Fat | 7851.87(2521.51) | 12749.44(2666.11) | 19861.83(5154.17) |
| Lean | 22306.35(4302.49) | 26533.57(4829.15) | 29800.64(5951.63) |
| **Subtotal** |  |  |  |
| BMC | 1814.25(415.38) | 2035.71(454.90) | 2115.68(482.57) |
| BMD | 1.00(0.12) | 1.04(0.12) | 1.05(0.13) |
| Fat | 16886.04(4686.32) | 24536.76(4962.48) | 37714.93(10071.58) |
| Lean | 41967.58(9142.91) | 50197.57(10039.97) | 56606.24(12168.67) |
| **Total** |  |  |  |
| BMC | 2310.62(448.52) | 2545.96(496.02) | 2619.31(527.91) |
| BMD | 1.13(0.11) | 1.17(0.12) | 1.18(0.12) |
| Fat | 17931.64(4671.63) | 25706.95(4938.64) | 38964.46(10094.93) |
| Lean | 44899.52(9417.73) | 53455.01(10328.62) | 60018.52(12576.19) |
| BMI, body mass index; BMD, body mineral density; BMC, body mineral content. Normal [BMI < 25 kg/m2], overweight [25 kg/m2 ≤ BMI < 30 kg/m2], obesity [BMI ≥ 30 kg/m2]. | | | |
